# Plant cultural indicators of forest resources from the Himalayan high mountains: implications for improving agricultural resilience, subsistence, and forest restoration

**DOI:** 10.1186/s13002-024-00685-w

**Published:** 2024-04-24

**Authors:** Shiekh Marifatul Haq, Aadil Abdullah Khoja, Muhammad Waheed, Andrea Pieroni, Manzer H. Siddiqui, Rainer W. Bussmann

**Affiliations:** 1 Department of Ethnobotany, Institute of Botany, Ilia State University, Tbilisi, Georgia; 2 Department of Botany, Glocal University, Saharanpur, U.P 247121 India; 3 University of Gastronomic Sciences of Pollenzo, Piazza V. Emanuele II 9, Pollenzo, 12042 Bra, Italy; 4 Department of Botany and Microbiology, College of Science, King Saud University, 11451 Riyadh, Saudi Arabia; 5 Department of Botany, Institute of Life Sciences, State Museum of Natural History, Karlsruhe, Germany

**Keywords:** Plant cultural indicator, Forest resources, Ethnic groups, Ecological transition-economic, Jammu and Kashmir, Indian Himalayan

## Abstract

**Aim:**

Biocultural legacy practices are intricately tied to forestry resources, ethnic identity, and social cohesiveness. This study aims to determine the plant cultural values of forest resources and identify plant cultural indicators in each ethnic group, which can aid in long-term natural resource management plans in the current debate on socio-environmental and ecological transitions.

**Methods:**

Semi-structured interviews, focus group discussions, and field observations were employed to collect data for a comprehensive and systematic ethnobotanical survey from February 2018 to October 2022.

**Results:**

A total of 330 informants reported 154 plant species from 65 families. Asteraceae was the most prominent botanical family, with herbaceous plant groups outnumbering trees and shrubs. The Gujjar and Pahari groups exhibited the highest level of overlap, followed by significant overlaps between the Gujjar and Kashmiri communities. The close affinity observed between the Gujjar and Pahari groups suggests the horizontal pattern of local plant knowledge between these communities, influenced by their sociocultural interactions and intermarriages. Notably, the Pahari community displayed a rich understanding of medicinal plants and shared unique uses for the reported taxa. This study affirms that both ecological factors and sociocultural influences have played significant roles in shaping local plant knowledge. A total of 31 plant species have been identified as plant cultural markers among all four ethnic groups. We observed a positive correlation between plant cultural values and plant use with the Gujjar and Kashmiri ethnic groups. *Artemisia absinthium* reported the highest use value of (0.57) with use reports of (189). *Adonis aestivalis, Cynoglossum nervosum, Geum elatum, Geranium himalayense, Juncus inflexus, Oxalis acetosella, Polygonatum biflorum,* and *Salvia hians* from the Himalayan region are among the plant taxa whose ethnomedicinal applications are described here for the first time.

**Conclusion:**

Our data show that local and indigenous forest knowledge and practices could significantly contribute to forest conservation and ecological transition. This may happen if stakeholders generate clear frameworks and biocultural conservation strategies aimed at both dynamically preserve natural habitats and ways of traditional management of local natural resources.

**Supplementary Information:**

The online version contains supplementary material available at 10.1186/s13002-024-00685-w.

## Introduction

Practices related to biocultural heritage are indissolubly entwined with forest resources, ethnic identity, and social cohesion [1]. Many communities in the Himalayan region rely heavily on forest resources as part of their survival strategy. Therefore, analyzing the use of forest products is essential for determining how human activity affects the environment and developing efficient conservation strategies [2]. What is done with and how much of the forest resources are harnessed depends on the socioeconomic status, resource availability, season, and traditional ecological knowledge (TEK) of the community [3]. Rural inhabitants, particularly those near forest regions, are more reliant on forest resource usage. The populations on the border of forests are very dependent on forests for their livelihood security, and among them, traditional knowledge of the diverse uses of plant species is passed down from generation to generation [4].

Exploring indigenous knowledge is necessary to ensure ecological, economic, and environmental sustainability [5]. Particularly populations on the edges of forests depend on forest resources to support their way of life. For better forest utilization, it is crucial to establish systematic and exacting data collection methods in all emerging nations [6]. Furthermore, the economic and social well-being of communities residing in and surrounding forest areas might be significantly improved if the government and stakeholders take the initiative to prioritize the sustainable use of forest resources [7]. Forest resources are at risk from the growing threat of widespread deforestation and habitat degradation [8]. Therefore, it is critical to quickly create adequate management methods and workable plans before these components are permanently lost. For the rehabilitation and maintenance of traditional and indigenous knowledge, these forest resources must be explored, used, and conserved by diverse ethnic groups [9].

In recent years, the field of Ethnobiology has garnered significant attention due to its exploration of the multifaceted influences of social, economic, ecological restoration, and political factors on local plant knowledge systems. Various studies have examined the impact of these drivers on different aspects, including the diverse ethnic groups [10], age demographics [11], socioeconomic conditions [12], religious beliefs [13], geopolitical transformations [14], and eco-restoration [15]. Biocultural heritage serves as the foundation for understanding the intricate connections between biological diversity and the language, cultural memory, ecological knowledge, and social values of local and indigenous communities [16, 17]. Recognizing the significance of biocultural heritage, it is evident that local communities hold valuable traditional knowledge that can contribute to both social and environmental sustainability [18, 19] as well as forest restoration efforts [20]. “Intangible cultural heritage is an essential pillar of cultural identity, particularly for minority and indigenous communities”. Within the realm of intangible cultural heritage, the perceptions use, and practices associated with the natural environment, including ethnoecological knowledge systems, hold considerable importance. Unfortunately, despite its significance in promoting food sovereignty, and social and environmental sustainability, ethnobotanical knowledge predominantly held by rural populations or practiced within community settings, has often been overlooked. To better understand scenarios where each plant has predictive ability over the species' cultural relevance, there is a need to multiply case studies that concentrate on looking for such characteristics. There is still more research to be done to determine if therapeutic or nutritional value is more important when choosing which wild plants are used. This study aims (1) to describe the extensive traditional knowledge of the local ethnic groups on the multipurpose use of forest resources in the targeted area, (2) to examine the cross-cultural overlap of forest resource utilization, and pattern of knowledge transmission among different ethnic groups, and (3) to determine the plant cultural values of forest resources and identify plant cultural markers in each ethnic group, which can aid in long-term natural resource management plans in the current debate on socio environmental and ecological transitions, (4) In addition, it would pinpoint the species that can be employed for extensive habitat restoration, agriculture resilience, livelihood, particularly in the context regional ecological and cultural significance.

By addressing these questions, we hope to highlight the value of ethnobotanical research in addressing issues relating to the current environmental, social, and economic challenges as well as the need for sustainable rural development to combat future calamities, i.e., biodiversity, poverty, and climate crises.

## Materials and methods

### Study area

**Fig. 1:**
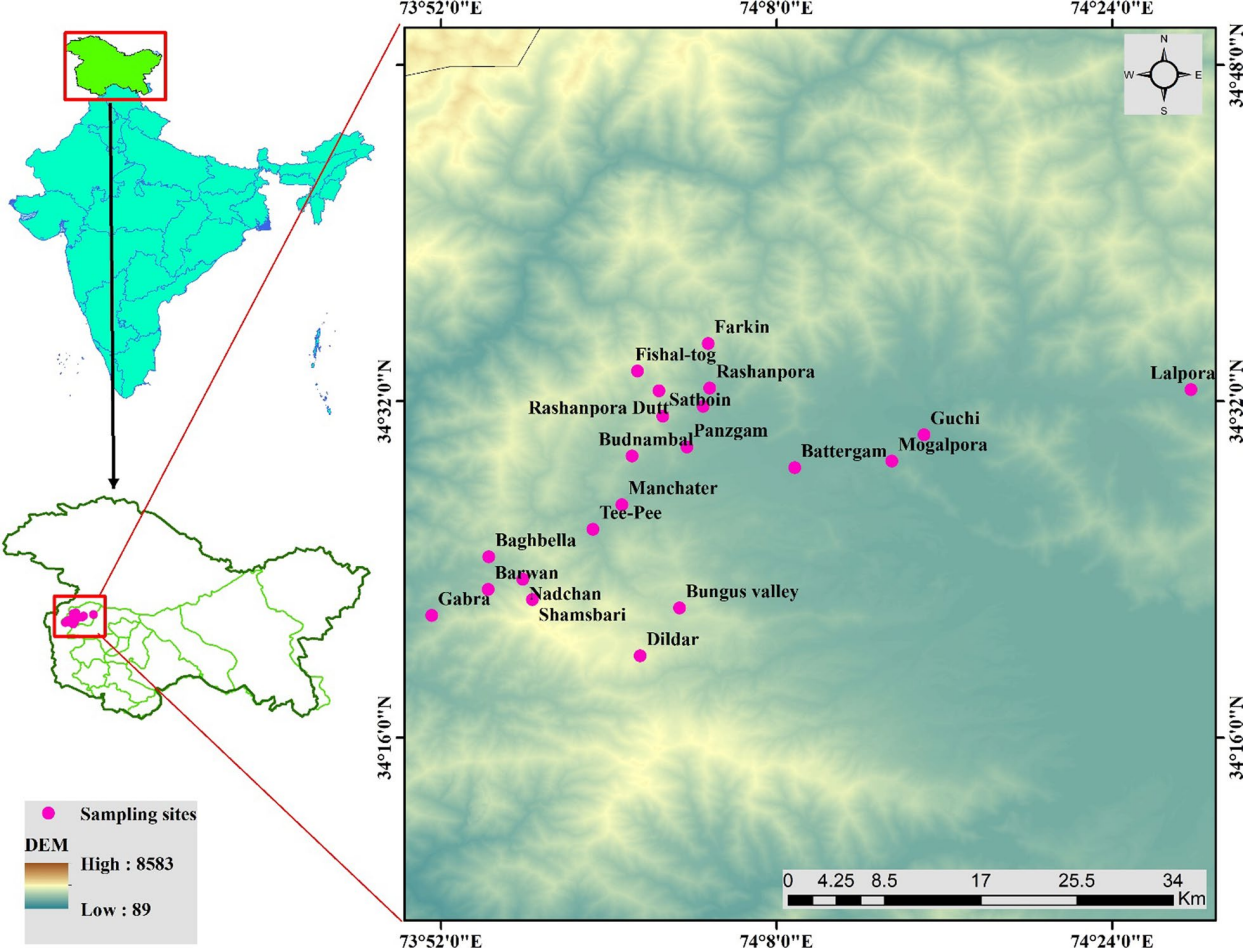
Map of the study area showing study sites in the district Kupwara of Jammu and Kashmir, India

Kupwara, situated in the northernmost region of the Kashmir Himalayas region, is a district that is home to 870,354 residents. The district spans 362 villages, with a population density of 368 individuals per square kilometer. (Available at https://www.census2011.co.in, accessed on June 11, 2021). It is located at 34° 31.5707′ N latitude and 74° 15.2768′ E longitude with an elevation from 1550 to 4073 m (Fig. 1). The district is situated between the Pir Panchal and Shams Bari Mountain ranges. The mountain ranges are flanked by pastures and meadows used for sheep and cattle grazing. Well-known passes like Sadhna Pass, Farkiyan Gali, and Nagmarg Pass cut through these mountain ranges. The passes provide access to the beautiful valleys of Karnah, Keran, and Drawa. The southern portions of Kupwara are primarily flat; the western, northern, and eastern belts of the district are hilly. Out of the total geographical area of 2379 km^2^ almost three-fourths are under dense forest cover, with the main forest types being Himalayan dry temperate, Fir Forest, and subalpine forest [21]. The area experiences a Mediterranean-style temperate climate, with winters being cold at higher elevations. The typical temperature is between 5 and 32 °C. The Kupwara district is known for its unique folklore, varied heritage, and rich culture and diversity. Most Kashmiris reside in the valley (Kashmir), along with Gujjars, Phari lives in low to high-altitude regions, Bakarwal (nomadic lifestyle). Kashmiris are frequently employed by the government, work in horticulture and agriculture, and are skilled/unskilled workers. The main connection between Gujjar, Phari, and Bakarwal is livestock rearing in the high-altitude regions. The Kashmiri ethnic group is half settled and settled tribes dwelling in lower to middle Himalayan ranges of the study area, while the Gujjar, Bakarwal, and Pahari ethic groups are half settled and unsettled ethnic groups, dwelling in lower to upper Himalayas of the study area.

### Data collection

The ethnobotanical survey was conducted in district Kupwara from 2018 to 2022. A snowball sampling method was used to select informants from different ethnic groups including Gujjars, Bakarwals, Kashmiris, and Phari. Open- and close-ended semi-structured interviews, as well as group discussions, were used to collect information [22]. To gather information regarding the traditional ethnobotanical uses of plant resources, including medicines, and their significance, a questionnaire was created. Furthermore, the focus of interviews and discussions was the ethnobotanical applications of local plant resources, including food, medicine, fuel, fodder, flavoring, and harvesting season information. The questions during the interview were: (1) Participants were asked about their demography details which include age, gender, ethnic group, occupation, etc. (2) What are the benefits you get from the forest? (3) Common name of the plants used? (4) Name commonly used medicinal plants? (5) Which plant part is used as medicinal plants? (6) How you prepare these medicinal plants? (7) What are the other uses of these medicinal plants? (8) In which season do you usually go for collection of medicinal plants? The detailed question asked during the survey is shown in Additional file 1. The research emphasis greatly influences the optimal approach for categorizing diseases and treatments using ethnomedical data, and it is important to translate from an emic to an etic perspective for effective biomedical screening of traditional treatments [23]. We used two methodologies in this study: (a) analyzing local and indigenous folklore practices and (b) looking for unusual flora with potential medicinal use.

The data were collected in different villages as illustrated in Fig. 1. To ensure accurate data collection, a translator was engaged to assist us in communicating with the residents of the selected villages, which are predominantly inhabited by the Gujjar, Phari, Bakarwal, and Kashmiri communities. The Bakarwal community, known for its nomadic lifestyle, visits the study area during June and primarily resides in the higher altitudes. On the other hand, Gujjars and Phari communities mainly reside in the study area but also migrate to higher altitudes during the summer season with their livestock. The Kashmiri community, meanwhile, represents the dominant population size in the study area.

To validate the collected information, we crosschecked it with relevant literature sources [24, 25]. Verbal prior informed consent was obtained from all the informants before conducting the interviews. Additionally, for each indigenous community, we selected an individual who was well-respected and knowledgeable about the traditions and norms of their respective community to guide us during the field surveys. Throughout the study, we adhered to the Code of Ethics of the International Society of Ethnobiology, which can be accessed at https://www.ethnobiology.net (accessed on 28 May 2022).

During our field studies, we collected detailed data on each plant specimen, including relevant taxonomic information. To identify the plants, we referred to taxonomic literature (https://efloraindia.bsi.gov.in/eFlora/eFloraHomePage.action). To preserve the collected plant specimens, we followed conventional herbarium procedures, drying, preserving, labeling, and pasting them onto herbarium sheets [26]. We updated the nomenclature using the Plants of the World Online (POWO) taxonomic database (https://powo.science.kew.org) and compared our plant specimens with those in the University of Kashmir Herbarium (KASH) collection. The voucher specimens were deposited in the KASH Herbarium, with field data incorporated into the herbarium sheets.

### Use value (UV)

The significance of the documented taxa was assessed by determining their use value (UV) [27]. The UV was calculated using the following formula:

$${\text{UV}} = \Sigma U/n$$

here ‘*n*’ represents the total number of participants in the study, while ‘*U*’ represents the number of reports in which each participant mentioned a specific plant taxon. The UV value for a species that was not mentioned ranged from 0 to 1. On the other hand, if a species was mentioned by every informant, its UV value was 1.

### Data analysis and disease categorization

A principal component analysis (PCA) was conducted to visualize the utilization of provisioning services and plant components. The function of fact was extra was used to illustrate the PCA biplot, contribution plot, and eigenvalues corresponding to the variance described by each principal component. To show the relation between life form, mode of preparation, and plant species chord diagram was prepared in Origin Pro software (version 9.95) [28]. We ascertain which species are linked to which life form and method of preparation, as well as the quantity of each species in each category, based on the thickness of each bar. Circular cluster heat map was constructed in Origin Pro software (version 9.95). Bioinformatics & Evolutionary Genomics software (http://bioinformatics.psb.ugent.be/cgi-bin/liste/Venn/calculate venn. http; viewed on 28 November 2022) was used to construct the Venn diagram of forest resources utilization between different ethnic groups. Through Venn diagram we were able to investigate cross-cultural similarities and differences of plant utilizations between ethnic groups. Nevertheless, the Venn diagram is unable to give an accurate representation of how plants are utilized; for instance, if a certain ethnic group employs N plants, it is not made apparent if this is the case for all informants or just a select few. For the first time, we employed indicator species analysis to resolve this issue. To determine the plant cultural values of forest resources and identify plant cultural markers in each ethnic group, we used Indicator Species Analysis using PAST software (version 4.12) [29, 30]. This gave information on the fidelity of a species to a certain group. Following the determination of each species' indicator values, which were modified from [28, 29], a Monte Carlo analysis was carried out to determine statistical significance. The relative abundance of a species in various cultural groups was determined based on the number of citations for each species from a distinct cultural group during indicator species analysis using the following formula:

1 $${\text{Relative}}\;{\text{abundance}}\left( {{\text{RA}}_{jk} } \right) = \frac{{x_{kj} }}{{\sum\limits_{k = 1}^{g} {x_{kj} } }}$$

where RA_*jk*_ means relative abundance, *x*_*kj*_ is the abundance of species *j* in group *k*, and *g* means the total number of groups.

2 $${\text{Relative}}\;{\text{frquency }}\left( {{\text{RF}}_{kj} } \right) = \frac{{\sum\limits_{i = 1}^{nk} {b_{{\dot{I}jk}} } }}{{n_{k} }}$$

where RF_*kj*_ is the relative frequency of plant *j* in group *k*, *b*_*ijk*_ is the presence or absence of plant *j* in group *k* sample *I*, and *I* is the sample unit.

3 $${\text{Indicator}}\;{\text{value}}\left( {{\text{IV}}_{Kj} } \right) = 100\left( {{\text{RA}} \times {\text{RF}}} \right)$$

To identify the indicator species, a threshold level of 25% indication and 95% significance (*p* ≤ 0.05) were used. Through this analysis we were able to identify the plant culturally significant species across specific ethnic groups. Our data made it possible to look at the relationships between the indicator values of many commonly used plants and the relative importance of species employed in ethnobotany and different ethnic groups.

## Results and discussion

### Respondents’ demography

The study area's local population continues to possess valuable traditional knowledge, which is evident from current research highlighting the strong connection between the local community and the provision of ecosystem services through forest resources. There were 330 respondents including ≈66% men and ≈34% women (Additional file 2). These respondents belonged to four different ethnic groups: Gujjars (*N* = 97), Phari (*N* = 88), Bakarwals (*N* = 77), and Kashmiri (*N* = 68), and represented a wide range of professional groups, including daily laborers, government workers, herders, shopkeepers, housewives, and herbalists. Women may be restricted to their houses due to cultural restrictions, which may account for the decreased proportion of female informants [31]. The greatest percentage of the informants (54.54%) were over the age of 56 years, followed by those between the ages of 28–55 (29.70%), and between the ages of 19–27 (15.76%). Over 50% of responders do not have any formal education. We found that older people held knowledge that was more conventional in this domain than younger people, which has also been found in other investigations [32].

It was noted that the illiterate population had more knowledge of traditional medicine, which may be explained by the fact that educated participants are expected to have exposure to the developed world and mostly rely on current medications rather than alternative one [33]. During the survey, it was discovered that people living in rural areas knew more about natural resources than people living in urban areas. In the rural area’s elders have higher traditional knowledge as compared to the younger once, the reason behind this is younger generation is not interested and have faith on tradition medicine as compared to generic medicine. This implies that the rural population is more connected to forest resources as compared to the urban population, elderly were the major caretakers of traditional knowledge, and if a structure is not put in place to ensure apprenticeship, the knowledge gap between the elderly and the young generation becomes a serious concern. In the current study, the link between locals and forest resources demonstrates the depth of indigenous knowledge on the various facets of plants used in the corridor. People's reliance on forest resources ranges from commonly utilized eating plants and medication to highly preferred fermentable plants.

### Diversity of forest species

In this investigation, 154 ethnobotanical plants from 65 families were documented, which have various uses, including medicinal, food, flavor, black magic, fuel, timber, and toxicity (Additional file 3). Regions with more diverse floras usually have more useful wild plant species for locals to report. The premise is that a broader choice of available species results in a correspondingly greater number being used [34, 35]. The way a species is used greatly depends on the socioeconomic conditions in the area, and distribution patterns might vary from location to location [36]. Families' contributions to different usage categories differed widely. The fact that several groups use forest resources shows how important these species are to the survival of the local community. The research revealed a notable dependence on a range of forest resources for medicinal applications. The research area had almost useful number of plant species as previous ethnobotanical investigations carried out in other Himalayan regions. In the trans-Himalayan region of Nepal, 121 and 116 species, respectively, were reported [37, 38]. From the Western Himalayas of Pakistan, more than 100 plant species were recorded in each study conducted previously [39] and from Kashmir Himalayan region less than 100 plant species were reported by [15, 22, 31, 32, 39–41]”.

Among the 65 botanical families, the gathered plant species were unevenly distributed and only nine families: Asteraceae (*N* = 18), Lamiaceae (*N* = 11), Ranunculaceae (*N* = 10), Polygonaceae (*N* = 6) Rosaceae (*N* = 6), Apiaceae (*N* = 4), Solanaceae (*N* = 4), Primulaceae (*N* = 4), and Plantaginaceae (*N* = 4) accounted for half of the reported plant species, while the other half belongs to 51 families (Additional file 3). Members of Asteraceae can adapt to arid and dry settings with ease because of their broad range of ecological amplitudes [22]. According to Kayani et al. [40], Ranunculaceae was classified as the most prominent family in the high-altitude areas of Pakistan. However, previous studies conducted in the Himalayan regions of India, Pakistan, and Nepal have indicated that Asteraceae is the dominant family [22, 32, 41]. Conversely, in the Eastern Himalayas, Lamiaceae is dominant [42].

### Life forms and plant part(s) used

Trees, shrubs, ferns, and climbers came in second in the current study after herb life forms for treating various illness categories (Additional file 3). The majority of highly valuable forest species were found at high altitudes, had a predominance of herbaceous form, and were utilized medicinally. Locals in the Himalayan region use large varieties of wild and non-cultivated edible plants for food, spice, fodder, and cultural purposes. Many plants are traded in great quantities to generate income, especially for their medicinal and aromatic properties. Wild plant consumption as food has been reported to be high in the Himalayan region year-round, but especially during the lean season. In addition to serving as a vital source of food and nutrition, forest plants play a significant role in Himalayan communities' culture and traditions. Forest resources that are accessible locally and are valued commercially have the potential to enhance the lives of rural mountain people. Through, careful planning the Phyto diversity of this region can considerably improve rural well-being by supporting health care and nutrition. Their understanding of plant habitats, availability, and the specific plant components they utilize significantly influence people’s utilization of forest resources. In forest-dwelling communities, the collection of forest plants is a common practice, driven by both the commercial exploitation of certain species and their demand from middlemen, who distribute them to markets beyond the local region.

**Fig. 2:**
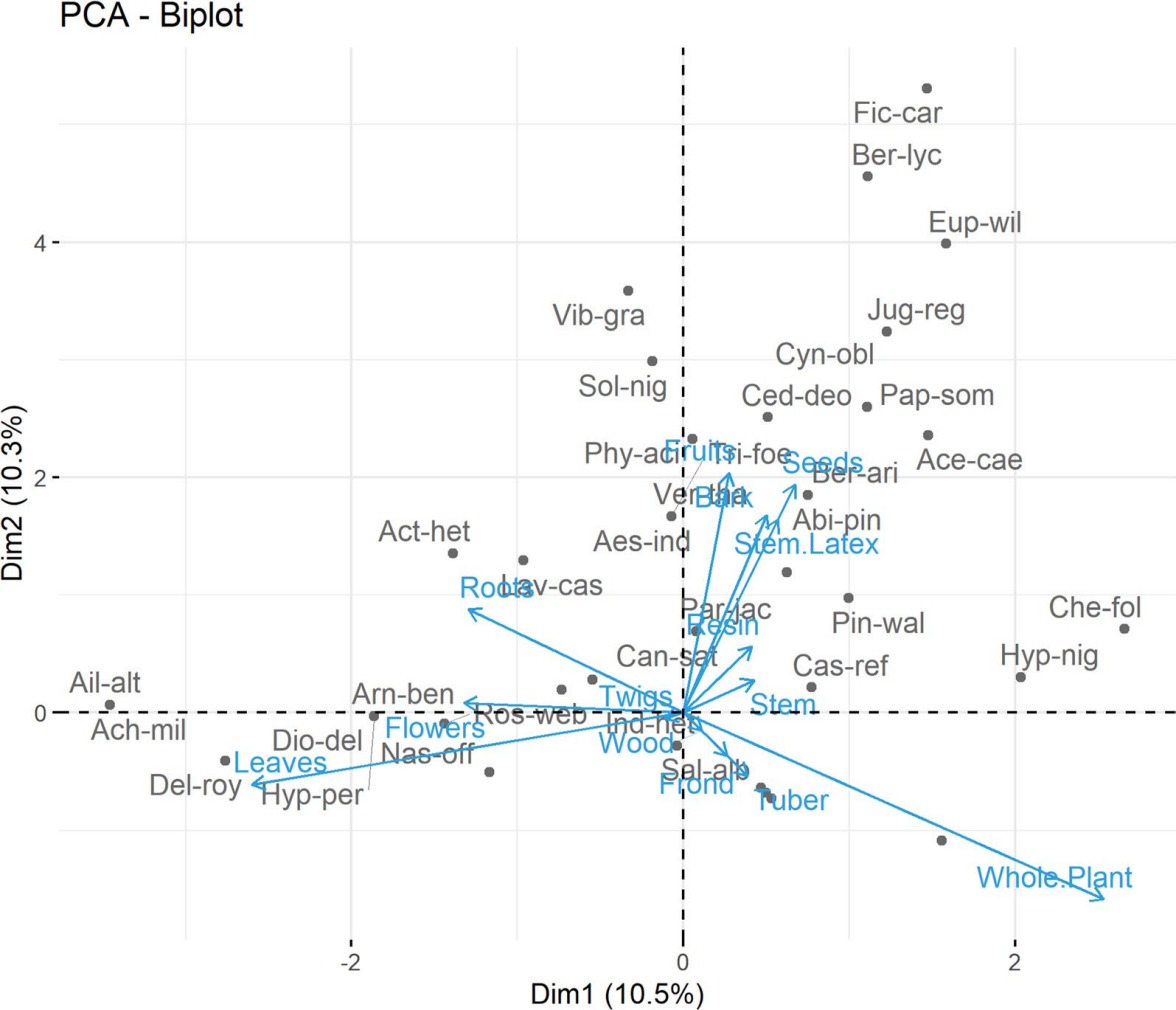
Principal component analysis (PCA) biplot of plant part(s) in the district Kupwara of Jammu and Kashmir, India

The local population employed numerous plant parts for ethnobotanical purposes and prescriptions made by traditional healers. Results of the preference analysis result from a significant variation, with leaves being most frequently used, followed by roots, whole plants, flowers, stems, latex, fruits, tubers, bark, resin, wood, seeds, and young twigs (Additional file 3). Most plant components were used to make homemade dry powders by crushing well-dried plant materials that were stored in glass bottles for further use. The PCA analysis identified fourteen distinct groups based on changes in the preferences for using plant parts. The biplot revealed 14 clusters of plant component usage based on species presence or absence, including leaves, roots, tubers, flowers, seeds, bark, whole plants, fruits, stem latex, stem, resin, wood, and young twigs. PC1 and PC2 described 20.8% of the parts utilized in the biplot (Fig. 2).

Herbs are abundant in nature and are particularly common in natural forests, along roadsides, and in-home gardens [43, 44]. Similarly, in the study area, the frequent use of herbs can be attributed to their easy accessibility from nearby forests. In addition, herbaceous species account for the majority of plant diversity in the Himalayan region's forests. It could be another reason that the majority of species identified for ethnobotanical usage came from the herb life form. Herbs play a vital role in forest ecosystems, particularly in the Kashmir Himalayas, and are utilized by local communities for their daily needs [15, 45]. Herbs, both annual and perennial, contain a considerable amount of bioactive chemicals and other secondary metabolites, which are highly effective in treating seasonal illnesses [46, 47]. Due to the dependence of indigenous communities on forest plants for their daily diet, different plant parts are favored based on their specific uses. Leaves, being the primary organs of photosynthesis, are known to be rich in metabolites [34, 48]. The use of leaves and aerial parts is considered sustainable and safe [49]. For therapeutic purposes, plant roots are often used or exchanged for goods by native pastoralists, herbalists, those involved in the herbal medicine trade, and people belonging to various ethnic groups. This tendency emphasizes how important the medicinal qualities of these plant-based ingredients are in conventional medical procedures. Plant roots are used and traded as essential components of the collective knowledge and customs of these societies, demonstrating a long-standing faith in the effectiveness of these botanical resources in treating a range of health issues [50]. Roots are recognized for their high concentration of bioactive substances [51]. However, it is important to discourage the overharvesting of underground parts or entire plants, particularly for vulnerable species, as this practice can lead to their eradication and decline in the wild [52].

### Preparations and disease categories

**Fig. 3:**
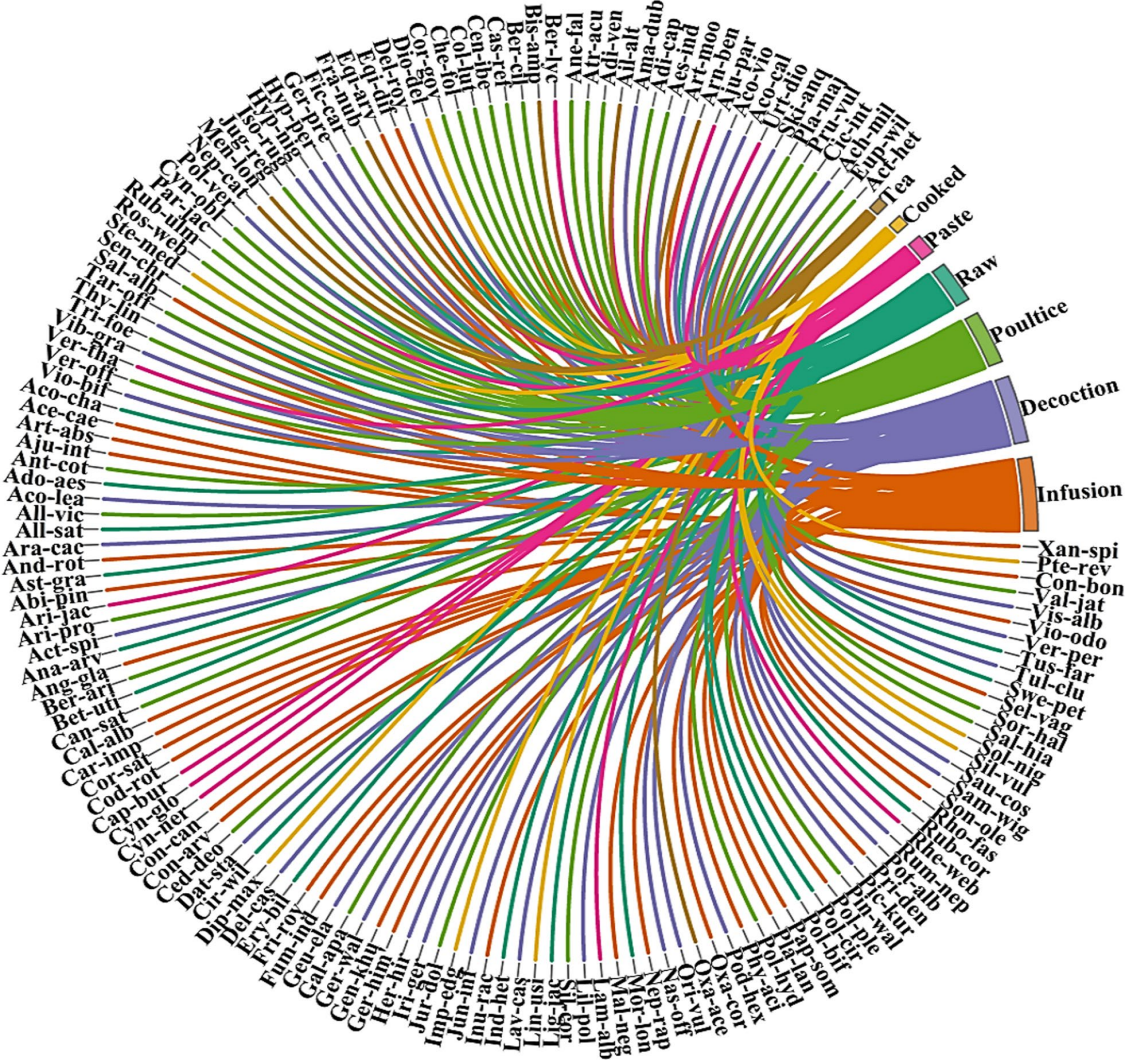
Distribution of plant species in various preparations used for the treatment of diseases. The direction of the lines depicts which plant species is linked with which preparation category and thickness of each bar indicates the degree of plants used to plant preparations in each category

**Fig. 4:**
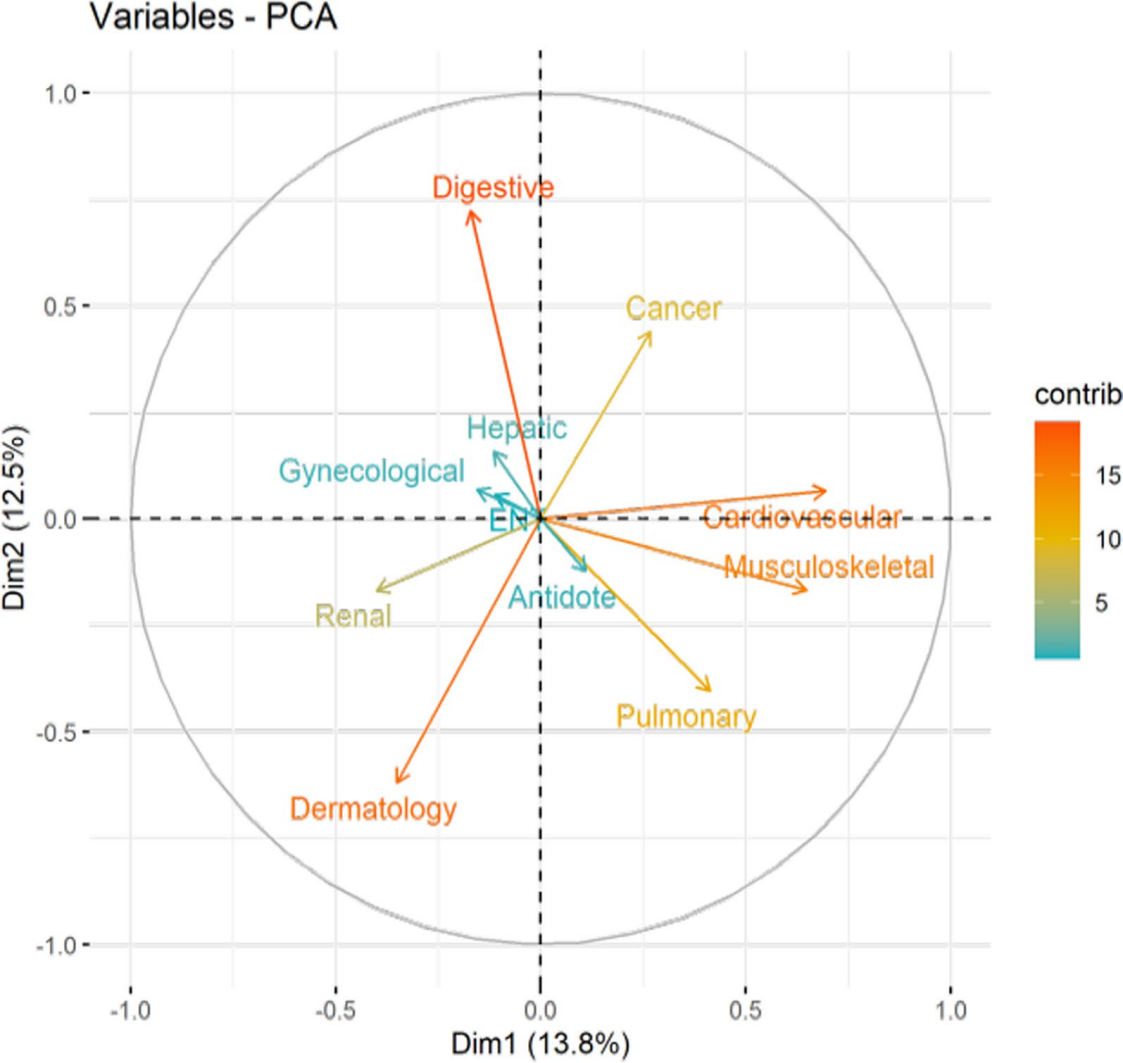
Principal component analysis (PCA) biplot of different disease categories in the district Kupwara of Jammu and Kashmir, India

The most common and viable strategies of traditional recipe preparations include using raw, drying the plants, crushing and grinding to fine powder, boiling to obtain decoction, making tea and infusion, poultice, cooked, and paste making (Additional file 3). As illustrated in Fig. 4, infusion (*N* = 58, 27%), followed by decoction (*N* = 53, 24%), poultice (*N* = 43, 19%), raw (*N* = 33, 15%), paste (*N* = 16, 7%), cooked and tea (*N* = 9, 4% each) were the most utilized preparations (Fig. 3). Most of the plant species are collected in the autumn season and depending upon the availability, forest inhabitants were aware of various plant species' collecting seasons, modes of collection, and frequency of collection. The preparations were commonly stored in glass bottles or other containers and utilized during the off-season or harsh winter conditions. Roots were the preferred plant part, as they tend to contain a higher concentration of bioactive constituents [51]. Aadil et al. [31] stated that grinding, boiling, and smashing were widely employed methods for extracting active ingredients in various regions worldwide.

The majority of the medicinal plant species were used to treat gastrointestinal diseases, colds, fever, sore throat, cough, dermatological infections, musculoskeletal disorders, respiratory system disorders, and nutritional abnormalities. Some of the commonly used medicinal plants were *Artemisia absinthium*, *Aconitum heterophyllum*, *Fritillaria roylei*, *Saussurea costa*, *Acorus calamus, Taraxacum officinalis*, *Jurinea dolomiaea*, *Rheum webbianum*, and *Geranium wallichianum, Capsella bursa-pastoris,* and *Cichorium intybus.* The specific usage categories were created while considering the use of various recognized plant-derived pharmaceuticals and medicines as well as the bodily system utilized. In the biplot (Fig. 4), PC1 and PC2 explain 26.3 percent of the disease categories. Eleven clusters of disease categories based on species presence/absence can be seen there: gastrointestinal issues, cardiovascular issues, dermatological issues, cancer, gynecological issues, pulmonary complaints (RES), musculoskeletal disorders, eyes, ears, and nose issues (ENT), hepatic diseases, renal disorders, and Antidote. Traditional knowledge is inextricably linked to local people's interactions with their resources and surroundings. However, the sustainability of this significant forest wealth received the least attention because forest resources were not prioritized by different forest policies until recently. Ethnic knowledge of the applications of many therapeutic plants was disappearing in the study area, as it was in other sections of the Himalayan region, due to the younger generation's lack of interest in passing down and applying ethnomedical practices [53].

### Other ethnobotany uses

**Fig. 5:**
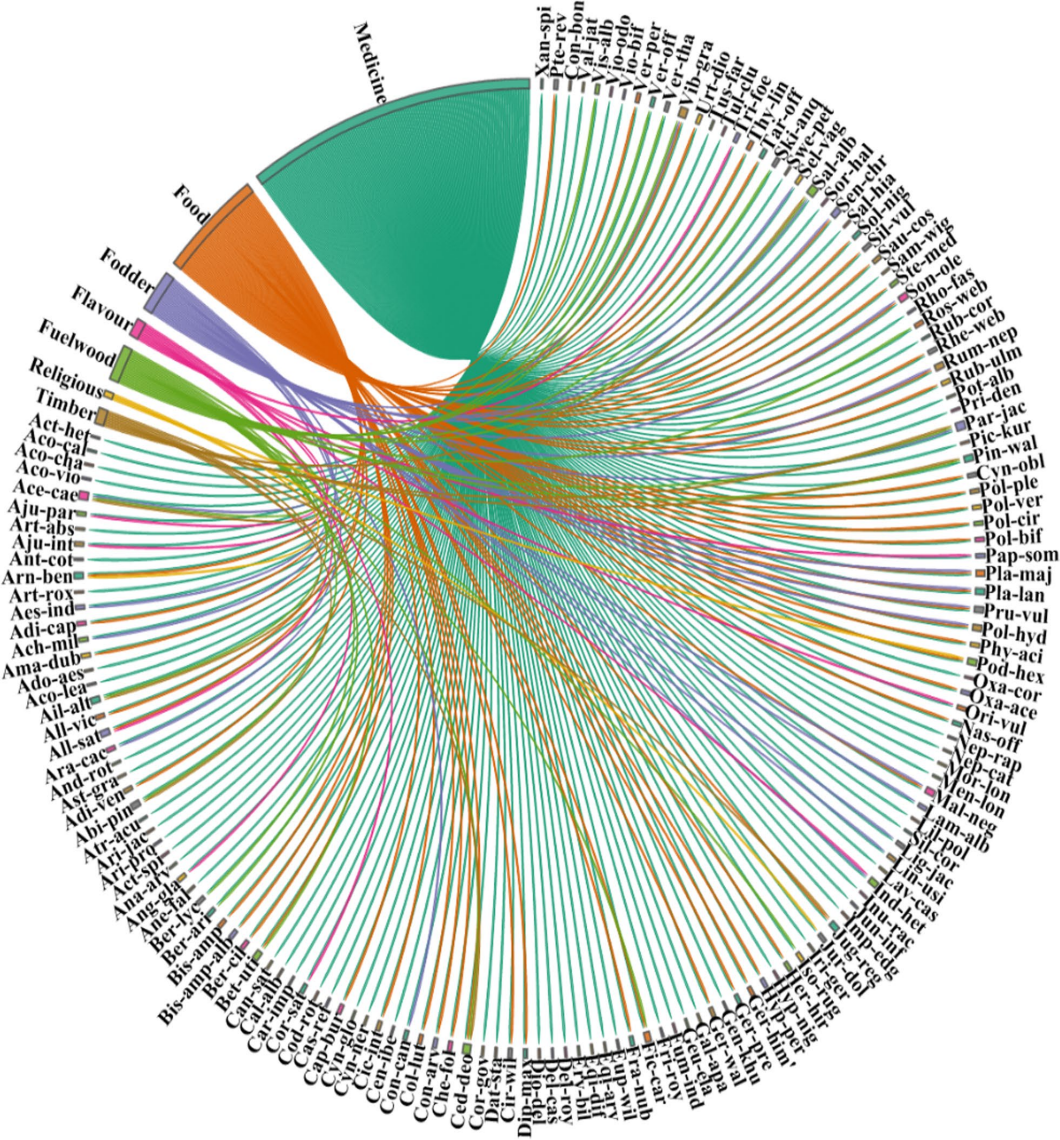
Distribution of plant species in various use categories in the region. The direction of the lines depicts which plant species is linked with which ethnobotanical usage, and thickness of each bar indicates the degree of plants used in each category

**Fig. 6:**
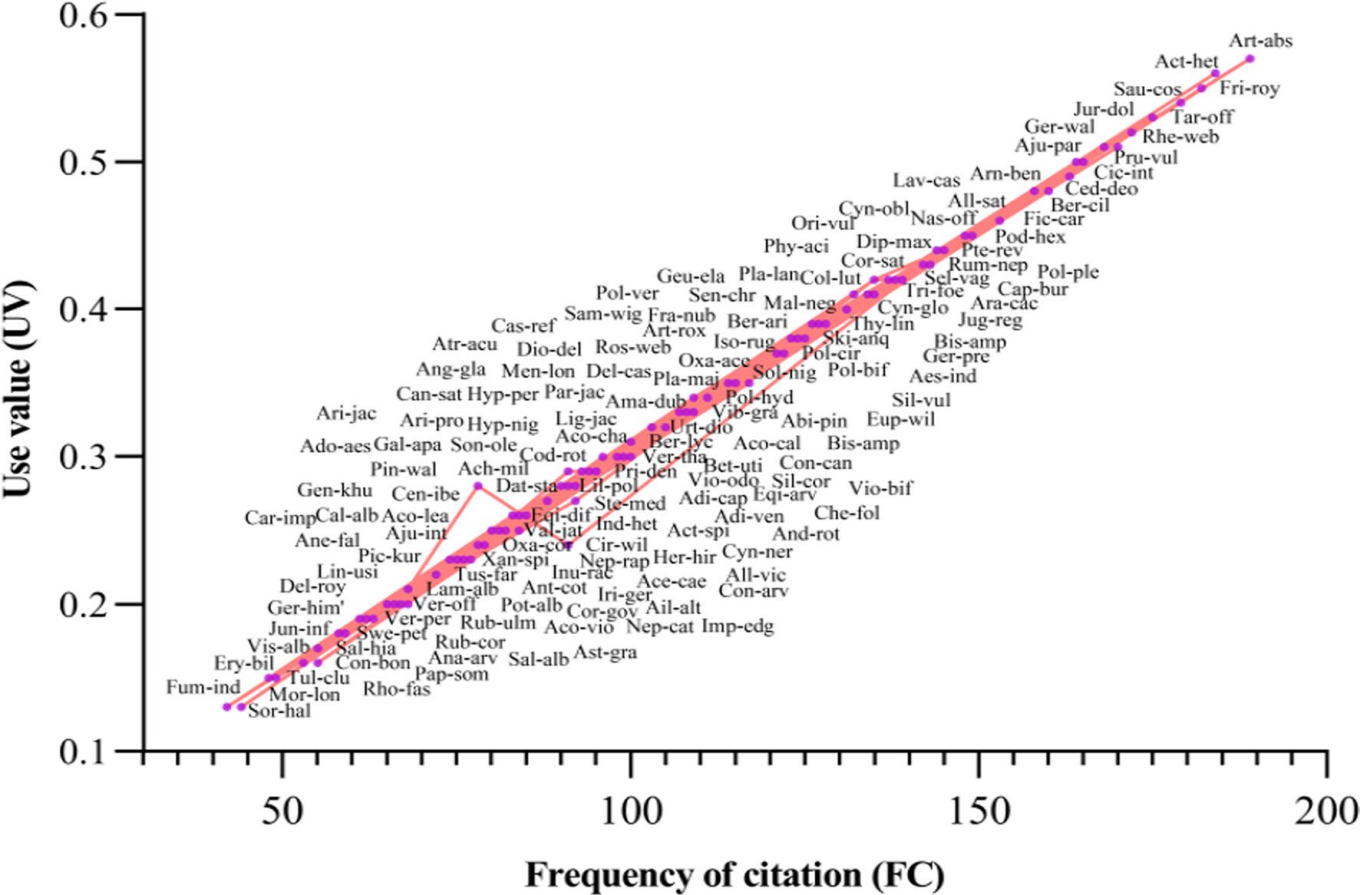
Relationship between use value (UV) and frequency of citation (FC). The full plant names are presented in Additional file 3

As shown in Fig. 5, more than half of the forest resources collected in the study area were used for medicinal purposes (57%), followed by food (21%), fuel wood (7%), fodder (8%), flavoring compounds, lumber (3% each), and religious applications (1%) (Fig. 6). Wild leafy vegetables growing adjacent to and in populated areas, such as *Amaranthus dubius, Allium victorials, Capsella bursa-pastoris, Cichorium intybus, Diplazium maximum, Malva neglecta, Nasturtium officinale, Prunella vulgaris, Plantago lanceolata, Pteridium revolutum, Rumex nepalensis, Rheum webbianum, Stellaria media, Silene vulgaris,* herbal tea is made from *Bistorta. amplexicaulis, Bergenia. ciliata, Betula utilis, Fragaria nubicola, Geranium. wallichianum, Geranium. pratense, Hypericum perforatum, H. hirsuta, O. acetosella*, and *T. linearis.* Some of the important plants that were used as fodder were *Acer caesium, Achillea millefolium, Conyza canadensis, Linum usitatissimum, Malva neglecta, Sonchus oleraceus, Salix alba, and Rumex nepalensis.* Fodder is mostly collected in the summer to autumn in the study area and it’s dried and stored for winter. Similar results were reported by [15, 54, 55]. Fuelwood plays an important role in the study area; some of the important fuelwood plants collected by the people of the study area were *Ailanthus altissima, Abies pindrow, Berberis lycium, Cedrus deodara, Parrotiopsis jacquemontiana*, and *Pinus wallichiana*. Plants used as flavoring agents were *Allium sativum, Angelica glauca, Coriandrum sativum, Origanum vulgare,* and *Trigonella foenum-graecum*. Some of the plants having religious uses like those that were used in religious gatherings were *Jurinea dolomiaea, Podophyllum hexandrum,* and *Arnebia benthamii.* Timber is used for construction purposes, handicraft in the study were some of the commonly used species were *Abies pindrow, Acer caesium, Juglans regia, Cedrus deodara, Pinus wallichiana,* and *Salix alba.* Species like *Aconitum chasmanthum, Acer caesium, Aconitum laeve, Atropa acuminata, Codonopsis rotundifolia, Datura stramonium, Delphinium cashmerianum, Euphorbia wallichii, Hyposcyamus niger, Phytolacca acinosa, Pteridium revolutum, Sambucus wightiana, and Saussurea costa* showed toxic effects*.*

### Use value

Ninety percent of the local respondents indicated that all the plants (154 taxa) they identified were used for self-care. The relative importance of botanical taxa used for treating particular types of ailments is indicated by the UV. The most important, well-liked, and valuable plant species that locals used as medicine were typically those with the highest UV. Such as *Artemisia absinthium* (0.57), *Aconitum heterophyllum* (0.56), *Fritillaria roylei* (0.55), *Saussurea costa* (0.54), *Taraxacum officinalis* (0.53), *Jurinea dolomiaea* (0.52), *Rheum webbianum*, and *Geranium wallichianum* (0.51 each) were the most popular plant taxa having UV above 50% (Fig. 6, Additional file 3).

The plant taxa with the greatest URs are those that were most collected for medical preparation because these medicinal taxa have multiple uses, i.e., medicinal as well as other uses. Therefore, it is important to prioritize their conservation and careful management to ensure their sustainable use. The taxa with the lowest UV values were *Juncus inflexus, Salvia hians, Veronica persica, Sorghum halepense, Potentilla alba* (0.12 each) *Fumaria indica* (0.13). Although these species were not well-liked by the locals, it has been observed that local healers regularly combined them with other plants in their regional herbal concoctions. According to the highest use report, there is a greater need for these medicinal plants to treat a variety of disorders, which raises their surplus demand and is the primary factor driving their extinction in their natural habitat. Multiple studies have highlighted the importance of certain plants in the Himalayas and their traditional medicinal uses among various tribal communities in the region. These studies provide evidence supporting the widespread utilization of these plant species as herbal medicines [56]. For example, *A. heterophyllum* and *A. absinthium* have been traditionally used by local communities to treat gastrointestinal illnesses, urinary infections, diarrhea, inflammation, digestive disorders, and high blood pressure [46, 54]. These plants contain phytochemicals such as lactones and terpenoids, which contribute to their medicinal properties [57]. The number of plant species employed in ethnomedicine corresponds to their overall utilization. One way to measure the relative usefulness of plants is through the use value (UV), which considers both the frequency of usage and the presence of these plants in literature sources [58]. It is important to note that a high UV value for a forest plant does not necessarily indicate numerous applications or mentions in publications [59].

### Cross culture-analysis

**Fig. 7:**
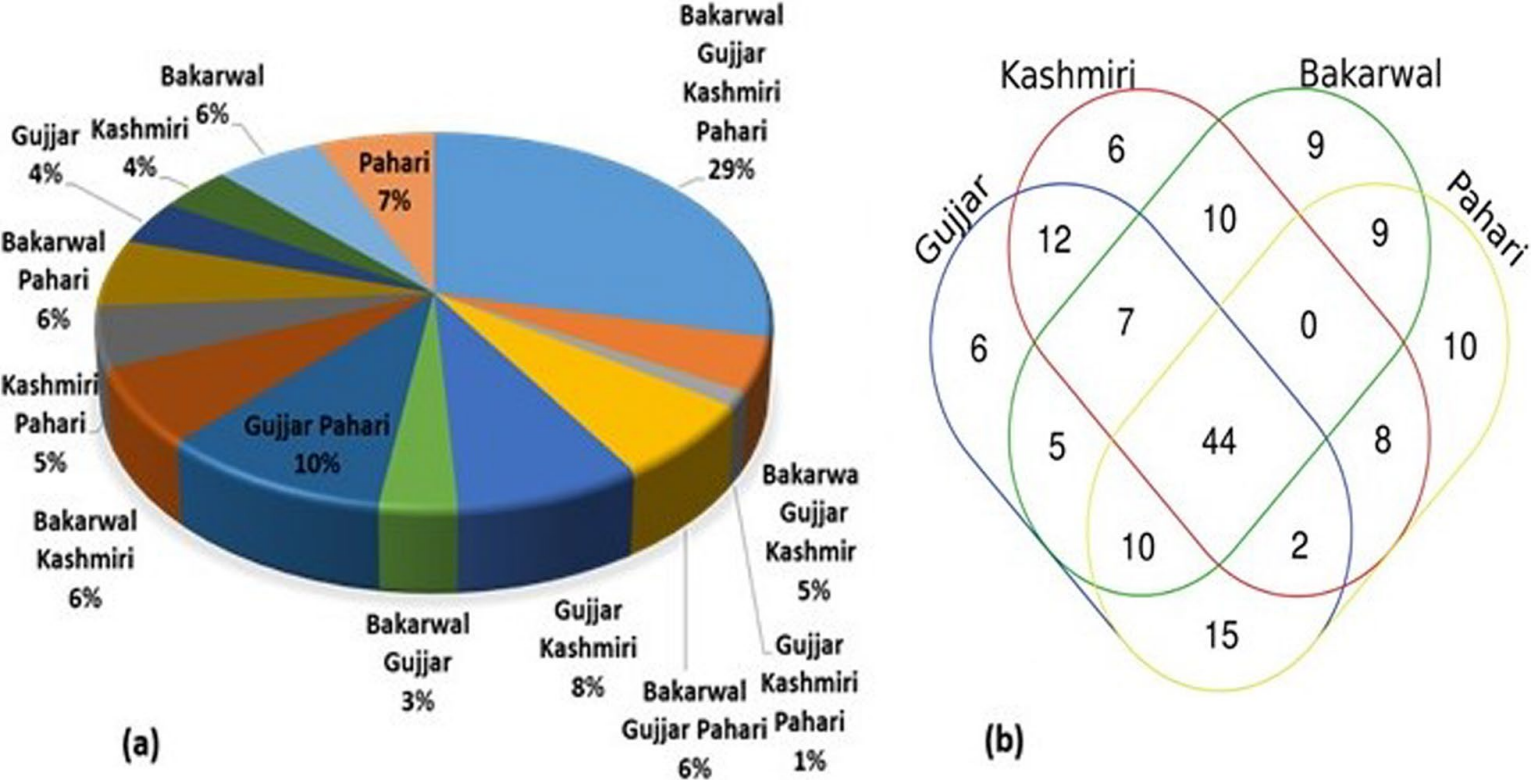
Venn diagram showing the overlap of use of plants by different ethnic groups in the district Kupwara of Jammu and Kashmir, India

Through cross-cultural analysis, it was evident that the ethnobotanical knowledge varied significantly among the four groups studied (Fig. 7a). Interestingly, we discovered that all four ethnolinguistic groups shared a common understanding of 44 plant uses, highlighting the diverse knowledge possessed by these communities regarding medicinal plants. It is hypothesized that because these forest species grow in the regions under similar natural environmental conditions, they benefit the health of the local population by preventing or treating common diseases in those same environments and climates. Although certain plants were popular because of their other ethnobotanical applications, most of the plants that were common to all cultures had medical, food, fodder, fuelwood, flavor, religious, and timber significance. The comparison of ethnobotanical knowledge among the four researched groups revealed varying degrees of similarity (Fig. 7b). The Gujjar and Pahari groups exhibited the highest level of overlap (10%), followed by significant overlaps between the Gujjar and Kashmiri communities (8%). Notably, the Pahari community displayed a rich understanding of medicinal plants and shared unique uses for the reported taxa. This study affirms that both ecological factors and sociocultural influences have played significant roles in shaping local plant knowledge. It is important to highlight the distinctiveness of the Gujjar and Pahari ethnic groups, as their medicinal ethnobotany and related practices stood out, although they had fewer idiosyncratic uses compared to other groups. The close affinity observed between the Gujjar and Pahari groups suggests the horizontal transfer of local plant knowledge between these communities, influenced by their sociocultural interactions and intermarriages. In contrast, the Bakarwal community, being predominantly pastoralists and having limited interaction with the other ethnic groups due to strict endogamy, knew fewer taxa and uses in their traditional medicine system. However, Bakarwal’s distinctive uses of plants indicated their extensive knowledge of species especially found in higher mountain areas, again reflecting their unique identity. The cultural isolation between the Bakarwal and Kashmiri groups has contributed to the preservation of specific interpretations and resource selection based on ethnic minorities and cultural differences. Their pastoralist traditions, preserved through cultural isolation, hold implications for adaptive strategies in the face of environmental changes. Furthermore, Bakarwal’s detailed observations can contribute significantly to conservation efforts and sustainable land management, with opportunities for integrating their traditional knowledge into broader initiatives for enhanced effectiveness and cultural relevance. Understanding and respecting Bakarwal’s unique perspective can enrich conservation practices and contribute to the preservation of biodiversity in the Himalayan highlands. Additionally, the study found that cross-cultural use of medicinal plants increased with altitude, indicating a preference for therapeutic plants from higher-altitude regions among the local inhabitants.

The traditional knowledge transfer mostly occurs between Gujjars, Phari, and Kashmiris. The transfer of traditional knowledge occurs in the downward direction, for instance, Gujjars and Phari transfer their traditional knowledge of medicinal plants to Kashmiris. As in the case of Bakarwals, the movement of traditional knowledge occurs from lower to higher regions. Just in the case of Bakarwals of other districts of Jammu and Kashmir (Rajouri and Poonch) move toward district Kupwara and transfer their traditional knowledge. The valuation of forest resources varies across different locations, as it is influenced by the local community’s perception of the quality, abundance, and exclusivity of plant species within their vicinity. A particular species may hold significant value in one location but not in another, depending on the needs and preferences of the people residing there. When a plant possesses strong cultural significance, it motivates the community to cultivate and utilize it for their future benefits.

Our cross-cultural analysis in the Himalayan highlands highlighted that different ethnic groups, including the Bakarwal pastoralists, still harbor outstanding knowledge of wild plants. This underlines the importance of revitalizing traditional ecological knowledge (TEK). The Bakarwals, as pastoralists, possess unique insights into pasture environments, likely due to their close and intricate relationship with these landscapes. Therefore, integrating TEK into the educational curriculum becomes crucial, especially considering the detailed understanding of the ethnic groups with natural resources. Initiatives such as workshops and seminars can play a pivotal role in sharing this specialized knowledge, helping not only preserve forest diversity but also the cultural diversity intrinsic to these communities. Our findings underline the need for recognizing culture as an integral part of biodiversity programs, emphasizing the symbiotic relationship between traditional practices and ecological sustainability. Revitalizing TEK represents a holistic approach to ensuring the preservation of both forest ecosystems and the unique cultural diversity observed in the Himalayan highlands, as highlighted by the distinct knowledge held by the Bakarwal community and other ethnic groups. The utilization of quantitative indices helps in assessing the importance of plant families within forest ecosystems, indicating that families rich in forest plants are more likely to be utilized compared to others. This observation aligns with findings from various regions. The use of such indices facilitates comparisons across different locations and cultural groups, enabling meta-analyses to be conducted. The cross-cultural use of Himalayan plants was also discussed by Haq et al. [10, 15] who found comparable results in the mountain ecosystem of the Himalayas.

### Plant cultural indicators

**Fig. 8:**
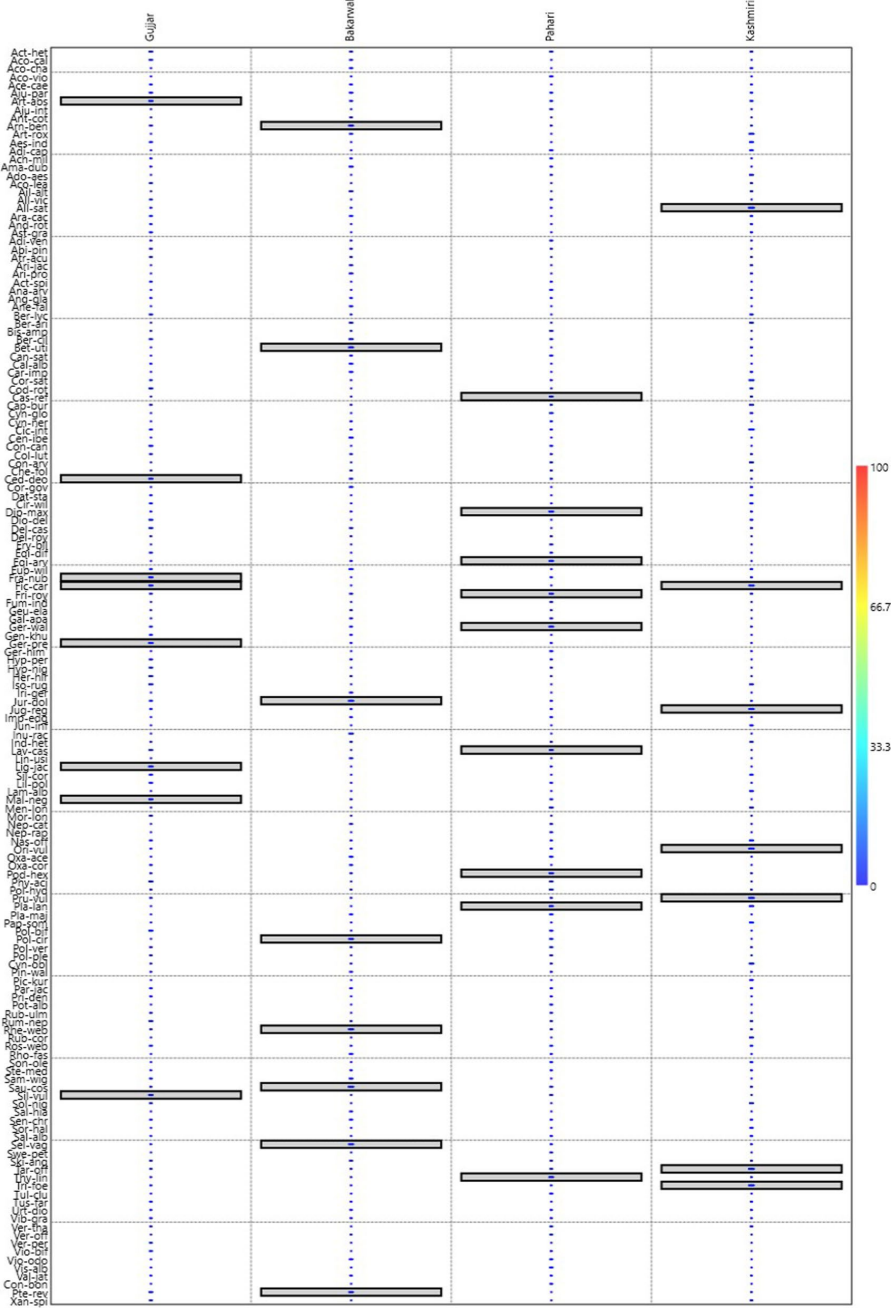
Indicator values of species in different ethnic groups in the district Kupwara of Jammu and Kashmir, India. Indicator plants are highlighted in box in different groups

Out of 154 plant species, a total of 31 plant species have been identified as plant cultural indicator species across all four ethnic groups. All the plant cultural marker species were reported based on multiple usages of plants like medicine, food, fodder, fuelwood, flavor, religious uses, and timber. A highest number of plant cultural marker species were reported by Pahari ethnic group (*N* = 9), and these were: *Capsella bursa-pastoris, Diplazium maximum, Equisetum arvense, Fritillaria roylei, Geranium wallichianum, Lavatera kashmiriana, Podophyllum hexandrum, Plantago lanceolata,* and *Thymus linearis* followed by Gujjar (*N* = 8): *Artemisia absinthium, Cedrus deodara, Fragaria nubicola, Ficus carica, Geranium pratense, Ligularia jacquemontiana, Malva neglecta* and *Silene vulgaris*, and Bakarwal (*N* = 7): *Arnebia benthamii, Betula utilis, Jurinea dolomiaea, Polygonatum cirrhifolium, Rheum webbianum, Saussurea costa,* and *Selinum vaginatum*, while Kashmiri also have (*N* = 7) *Allium sativum, Ficus carica, Juglans regia, Origanum vulgare, Prunella vulgaris, Taraxacum officinalis*, and *Trigonella foenum-graecum* plant cultural marker species (Fig. 8).

*Ficus carica* was the only plant cultural marker species that was represented by two ethnic communities, i.e., Gujjar and Kashmiri. The reason behind this is the multiple uses of this species. Mostly it’s used as a fruit tree having highly medicinal values as well as used as timber. The Pahari group reported the highest number of culturally significant plant species, which can be attributed to their sociocultural interactions. Their close relationships, intermarriages, economic and traditional negotiations, and sharing the same language with other ethnic groups have likely contributed to this. The Pahari ethnic group, being involved in animal husbandry, possesses significant traditional ecological knowledge of natural resources. They have a strong connection with nature, primarily due to their socioeconomic status, as they are predominantly poor and heavily reliant on traditional medicine. Other cultural marker species like *Diplazium maximum, Fritillaria roylei, Geranium wallichianum, Podophyllum hexandrum, and Plantago lanceolata* also have multiple along with medicinal and food values. The Gujjar and Bakarwal ethnic groups have reported eight and seven plant cultural marker species, respectively. This can be attributed to their cultural connections and shared language, as well as the increased population density in the research area. The knowledge exchange resulting from cross-marriages and social contacts, along with their occupations closely related to traditional medicines, likely contribute to their higher knowledge of plant species. The diverse sociocultural variations among these ethnic groups spread across different geographical regions in the study area may explain the variations in reported plant uses. It’s important to note that plant cultural marker species used by the Bakarwal ethnic communities were *Arnebia benthamii, Betula utilis, Jurinea dolomiaea, Rheum webbianum, and Saussurea costa* which are present at higher altitudes of the study area and easily available to the Bakarwal ethnic group. The least number of plant cultural marker species was reported by the Kashmiri ethnic group (*N* = 7); the reason for this may be possibly because they live in urban environments and are more exposed to generic medicine as well as a decrease in tradition as well as ethnomedicinal knowledge. Plant cultural marker species in the Kashmiri ethnic group were commonly used cultivated plants *Allium sativum, Ficus carica, Juglans regia, Organum vulgare,* and *Trigonella foenum-graecum.* All these plant species were grown in home gardens because they were being used in everyday life apart from their medicinal values.

### Classification of cultural plants

**Fig. 9:**
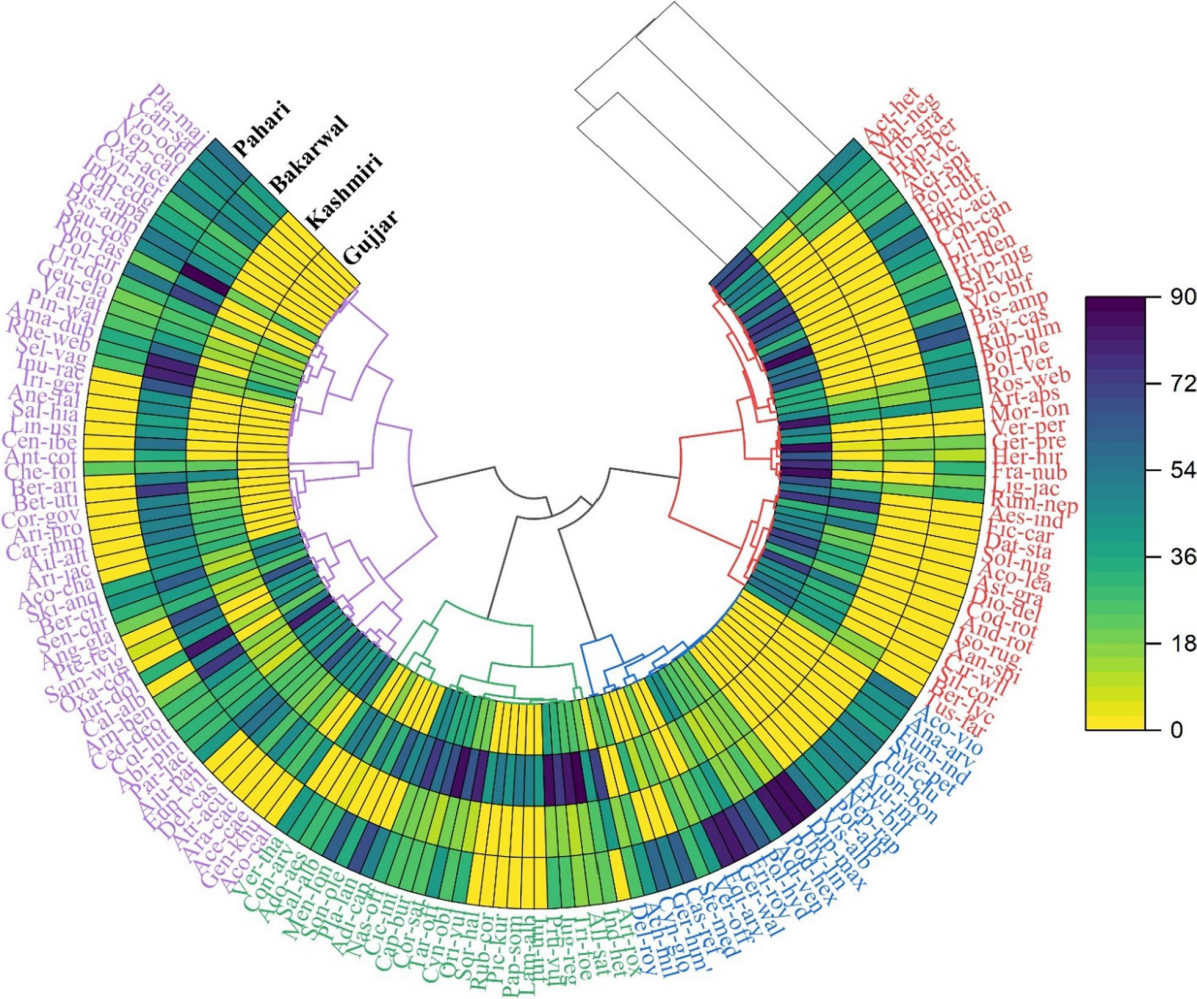
Classification of medicinal plants on the basis of number of citations in different cultural communities

The recorded plants were classified into four groups based on several citations through the Bray–Curtis distance method. Group 1 included 44 species, and more citations were seen in the Gujjar community followed by the Pahari community. The plant which has the highest number of citations for the Gujjar community include *Aconitum heterophyllum, Malva neglecta, Polygonatum biflorum, Phytolacca acinosa, Conyza canadensis, Silene vulgaris, Artemisia absinthium, Geranium pratense, Ligularia jacquemontiana,* and *Fragaria nubicola*. dominant includes only two highly cited species *Ficus carica* and *Solanum nigrum* in group 1 (Fig. 9). Group 2 includes 26 species with the highest citations in Pahari communities. The highly cited species by the Pahari communities in this group include *Diplazium maximum, Thymus linearis, Podophyllum hexandrum, Geranium wallichianum, Equisetum arvense, Polygonum hydropiper, Fritillaria roylei.* Group 3 included 27 medicinal plants with the highest citations in the Kashmiri community. Notable species which has the highest number of citations were *Allium sativum, Trigonella foenum-graecum, Juglans regia, Prunella vulgaris, Origanum vulgare, Taraxacum officinalis, Capsella bursa-pastoris,* and *Cichorium intybus*. In the Pahari community, only two species have higher citations in this group including *Menthe longifolia* and *Plantago lanceolata*. Group 4 includes 57 species with the highest citations shown by the Bakarwal community. The plant with higher citations for Bakarwal community include *Arnebia benthamii, Jurinea dolomiaea, Betula utilis, Selinum vaginatum, Inula racemose, Rheum webbianum, Amaranthus dubius, Polygonatum cirrhifolium*, and *Saussurea costa*. Most of the plants included in this group were cited by Bakarwal and Pahari communities. The highly cited plants by the Gujjar community included *Cedrus deodara, Acorus calamus,* and *Delphinium cashmerianum*. The plants with higher citations in the Pahari community included *Plantago major, Viola odorata, Cannabis sativa, Oxalis acetosella,* and *Bistorta amplexicaulis*. Khoja et al. [54] also reported *Aconitum heterophyllum, Malva neglecta*, and *Polygonatum biflorum* are highly utilized plants for the Gujjar community in Kashmir Himalaya. Similarly, Ridwan et al. [60] documented *Podophyllum hexandrum* and *Diplazium maximum* in Pahari community in Rajouri district. Trak and Giri [61] reported *Arnebia benthamii, Jurinea dolomiaea, Betula utilis, Selinum vaginatum, Rheum webbianum,* and *Polygonatum cirrhifolium* in the Gujjar and Bakarwal communities of district Kishtwar.

### Novelty

In the current study, 43 different types of diseases were reported for the first time, different medicinal plants treated those. A few of the examples were; the treatment of tonsillitis by *Aconitum heterophyllum*, cough and cold by *Acorus calamus*, flowers of *Achillea millefolium* as a brain tonic, leaves of *Ailanthus altissima* as wound healing agents, seeds of *Cydonia oblonga* in the treatment of boils and stem latex of *Euphorbia wallichii* in the treatment of foot corns. Out of different medicinal preparations used in the study area (*N* = 38) were reported for the first time in the study area. Some plant parts that were not previously reported in the study area were dried leaves *Viola biflora* were boiled in water and the extract taken orally after cooling, Seeds of *Viburnum grandiflorum* were boiled in water for 20–25 min and the extract was taken thrice a day for 5 days, *Mentha longifolia* of whole plant is cooked with egg and taken orally and fruits of *Ficus carica* boiled in water and kept overnight and taken orally early in the morning. Out of different plant parts used in the study area (*N* = 22, 33.88%) were reported for the first time in the study area. Some plant parts, which were not previously, reported area of seeds of *Aconitum heterophyllum*, leaves of *Arnebia benthamii*, the bark of roots of *Berberis lyceum*, whole plant of *Chenopodium foliosum,* and flowers of *Delphinium roylei*.

### Threats to plant species and traditional knowledge

This study showed that growing modernization and globalization cause more than just the erosion of traditional knowledge; they also make exploration and accessibility simple. This raises the demand for significant medicinal plant species and propels the discovery of molecules with pharmacological applications. Critically endangered and rare important medicinal plant species found in the region were, e.g., *Aconitum heterophyllum, Aconitum chasmanthum, Aconitum benthamii, Aconitum victorials, Berberis aristata, Betula utilis, Delphinium cashmerianum, Fritillaria roylei, Gentiana khuroo, Inula racemosa, Lilium polyphyllum, Podophyllum hexandrum, and Saussurea costa.* Overuse or overharvest of these species endangers biodiversity and the community’s capacity to use them for traditional medicine. Overharvesting of subsurface components or entire plants should be avoided, especially when it comes to endangered species, as this practice reduces the plant's wild population [15, 52].

### Implications for improving agricultural resilience, subsistence, and forest restoration

We identified 32 plant cultural indicators across four ethnic groups based on medicinal usage as well as other ethnobotanical uses such as food, fodder, and fuel wood. The Pahari ethnic group reported the most plant cultural indicators (*N* = 9), indicating their strong relationship with natural resources; however, the Kashmiri ethnic group had the fewest (*N* = 7), indicating the eroding of traditional knowledge in this group. Furthermore, we have observed that TEK is gradually disappearing among many mountain communities because of socio environmental transitions, and thus ethnobotanical studies devoted to recording the disappearing knowledge on important plant cultural markers could represent a remarkable resource to combat future calamities. Species like *Capsella bursa-pastoris, Plantago lanceolata, Malva neglecta, and Taraxacum officinalis* are adapted to climatic and environmental conditions and by incorporating these plant marker species into conventional agriculture; food production systems may become more resilient and sustainable. Many species like *Prunella vulgaris, Malva neglecta*, *Fragaria nubicola, Plantago lanceolata, Thymus linearis, Trigonella foenum-graecum*, *Diplazium maximum,* and *Taraxacum officinalis* grow in wild and could additionally become a part of home gardens to overcome food security issues soon. For marketing activities, local knowledge is a valuable resource. For local communities, species like *Fritillaria roylei, Saussurea costa, Rheum webbianum, Lavatera kashmiriana, Podophyllum hexandrum, Jurinea dolomiaea*, and *Arnebia benthamii* provide considerable income. However, due to globalization, the demand for these precious resources has increased, which has resulted in the extinction of some of these species. To ensure the long-term viability of these priceless resources, we advise the reintroduction or recovery of these species through community involvement. For young students in the area to create an emotional connection with the environment, they must be well-informed about the ecological and cultural significance of local natural resources from a young age. They might also have a stronger impact on bringing regional sustainable practices to the attention of the public. Existing local knowledge can be used to guide forest management in the face of future climate change. The multiple plant cultural markers identified can be used to boost species recovery, such as modifying structure through enhanced species regeneration and planting native species for instance by planting and reseeding species (*Cedrus deodara*, *Betula utilis, Arnebia benthamii, Fritillaria roylei, Geranium wallichianum, Lavatera kashmiriana, Podophyllum hexandrum, Ligularia jacquemontiana, Silene vulgaris*, *Jurinea dolomiaea, Polygonatum cirrhifolium, Rheum webbianum, Saussurea costa,* and *Selinum vaginatum*). Our study highlights that species like *Capsella bursa-pastoris, Equisetum arvense, Fargaria nubicola, Thymus linearis, Artemisia absinthium, Malva neglecta Geranium pratense, Prunella vulgaris, Taraxacum officinalis, Organum vulgare, Plantago lanceolata, and Ficus carica* tolerate extreme climatic and environmental conditions and indicate that these species may be used as pioneer species particularly degraded landscape modifying the habitat for other species, thus be promoted for future forest restoration in the landscape particularly denuded area. Certain edible cultivated plants like *Ficus carica, Juglans regia, Trigonella foenum-graecum*, *and Allium sativum,* and their constituents have been associated with a reduced risk of diseases, so we encourage their production. In the current debate on ecological transition, policymakers must articulate appropriate measures to avoid the devastating impact of socio environmental change on mountain forest recourses. To achieve this, collaborative efforts must focus on knowledge exchange, and respecting and integrating indigenous wisdom into conservation strategies. Documenting and archiving this knowledge ensures its longevity, fostering a sense of empowerment within the local communities. Policies must uphold cultural isolation to maintain distinct ethnobotanical practices. Ultimately, incorporating traditional wisdom into conservation initiatives will contribute significantly to preserving the biocultural diversity in the Himalayan highlands.

## Conclusions

This study found that numerous ethnic groups in the Himalayan highlands have outstanding knowledge of wild plants. The study also showed that each community has managed to preserve its own unique traditional ethnobotanical knowledge. Comparatively speaking, the Pahari ethnic group had a greater retention of knowledge than the Kashmiri ethnic group. The uniqueness of the data shows that human ecological characteristics have been crucial in the transfer of knowledge. Like how sociocultural communication has been essential in spreading local knowledge across ethnic groups. Furthermore, the cultural isolation between the Bakarwals and Kashmiri groups has assisted local communities in retaining their specific interpretations of natural resource use, indicating that these Bakarwals groups have their special selection of resources based on the unique composition of ethnic minorities and culture. We identified 32 plant cultural markers across four ethnic groups that have the significant ecological and cultural value of a plant, which will encourage the public to plant to gain benefits soon. Thus, the reintroduction or recovery of these species should be promoted through community involvement based on traditional ethnobotanical knowledge to preserve the distinct transfer of knowledge. This marriage between indigenous ecological knowledge and bio-conservation efforts could have also relevant benefits for fostering sustainable uses of marketable NTFPs, mitigating climate change adaptation, sustaining common forms of management of natural resources, and also revitalizing TEK cultural heritage among younger generations, via appropriate educational platforms. Overall, our study shows that local and indigenous forest knowledge and practices offer valuable insights and also a few possible solutions for addressing contemporary conservation and ecological challenges. Integrating these traditional perspectives with scientific knowledge can lead to more comprehensive and effective strategies for forest conservation and ecological transition.

### Supplementary Information

**Additional file 1.** List of Questionnaires for data collection.

**Additional file 2.** Demographic details of respondents.

**Additional file 3.** List of plant species, local name, part used; life form; preparation, diseases treated, other ethnobotanical uses, traditional cultural use across the four ethnic groups from the Western Himalayas.

## Data Availability

All the data are available in the manuscript.

## Abbreviations

FC: Frequency of citation
NTFPs: Non-timber forest products
POWO: Plants of the world online
PCA: Principal component analysis
TEK: Traditional ecological knowledge
KASH: University of Kashmir Herbarium
UV: Use value
