# Supplementary material for: Plant cultural indicators of forest resources from the Himalayan high mountains: implications for improving agricultural resilience, subsistence, and forest restoration
Source: J Ethnobiol Ethnomed. 2024 Apr 24;20:44. doi: 10.1186/s13002-024-00685-w (PMC11040985; doi:10.1186/s13002-024-00685-w)
Supplement: Supplementary file 1 — Additional file 1. List of Questionnaires for data collection. [file 13002_2024_685_MOESM1_ESM.docx]

**Name of respondent: Location**

1. **Gender**
2. **Age**
3. **From which ethnic group you belong?**
4. **What is your highest educational qualification?**

- Graduate and above ( )
- Intermediate ( )
- Matriculation ( )
- 8^th^ pass ( )
- 5^th^ pass ( )
- Illiterate ( )

1. **What is your main** **occupation?**

- Unemployed ( )
- Student ( )
- Cultivator/Agricultural laborer ( )
- Non-agricultural laborer ( )
- Daily wage laborer ( )
- Domestic servant ( )
- Skilled/semi-skilled worker ( )
- Large business/self-employed ( )
- Other ( Specify )

1. **What are the benefits you get from the forest?**

- Food (plant based)
- Timber
- Fuelwood and fodder
- Medicinal plants
- Any other (specify).

1. **Common name of the plants used?**
2. **Which plant part is used as medicinal plants?**

- **Whole plant**
- **Leaves**
- **Roots**
- **Fruits**
- **Bark**
- **Flower**
- **Stem**
- **Tubers**
- **Stem latex**
- **Twigs**
- **Resin**
- **Wood**
- **Seeds**
- **Other**

1. **How you prepare these medicinal plants?**

- **Raw**
- **Powder**
- **Infusion**
- **Decoction**
- **Tea**
- **Cooked**
- **Paste**
- **Poultice**
- **Other**

1. **Diseases treated?**
2. **Name commonly used medicinal plants?**
3. **What are the other uses of these medicinal plants?**

- **Food**
- **Fodder**
- **Fuelwood**
- **Timber**
- **Flavor**
- **Black magic**
- **Toxicity**

1. **In which season do you usually go for collection of medicinal plants?**

- Spring (March-April)
- Summers (May-July)
- Autumn (Aug to Oct)
- Winters (Nov-Feb)
- All season

1. **Name some commonly used medicinal plants?**
2. **What is the source of knowledge?**

- Folklore
- Radio/TV
- Accidently
- Ayush/hakim
- Books
- Any other (specify).

1. **Do you preserve these medicinal plants: Y/N (Duration…………….)**
2. **What is the used of collected medicinal plants?**

- House hold use.
- Sold

**Place:**

**Date:**

**Time:**

**Geographic coordinates:**
