# Supplementary material for: Plant cultural indicators of forest resources from the Himalayan high mountains: implications for improving agricultural resilience, subsistence, and forest restoration
Source: J Ethnobiol Ethnomed. 2024 Apr 24;20:44. doi: 10.1186/s13002-024-00685-w (PMC11040985; doi:10.1186/s13002-024-00685-w)
Supplement: Supplementary file 2 — Additional file 2. Demographic details of respondents. [file 13002_2024_685_MOESM2_ESM.docx]

**Additional file 1.** Demographic details of respondents.

| **Demography** | **Total/ Percentage** | **Ethnic groups** | | | |
| --- | --- | --- | --- | --- | --- |
|  |  | Pahari | Bakarwal | Kashmiri | Gujjar |
| **Respondents** | 330 | 77(23.33%) | 68(20.60%) | 97(29.39%) | 88(26.67%) |
| **Male** | 218(66.06%) | 30(13.8%) | 22(10.1%) | 33(15.1%) | 33(15.1%) |
| **Female** | 112(33.94%) | 23(20.5%) | 21(18.8%) | 28(25%) | 25(22.3%) |
| **Original Language** |  | Pahari | Gujari | Kashmiri | Gujari |
| **Tribes** |  | Half settled & unsettled | Half settled & unsettled | Half settled & settled | Half settled & unsettled |
| **Distribution** |  | Lower to Upper Himalayan range | Lower to Upper Himalayan range | Lower to Middle Himalayan range | Lower to Upper Himalayan range |
| **Livelihood source** |  | Horticulture, Cattle rearing | Pastoralism | Horticulture, Cattle rearing | Horticulture, pastoralism |
| **Religion** |  | Shia and Sunni Islam, Hinduism | Sunni Islam | Shia and Sunni Islam | Sunni Islam, Hinduism |
| **Origin** |  | Indigenous Group of Himalayas | Gurjara kingdom (570ce) | Indo-europena ethno-linguistic group | Gurjara kingdom (570ce) |
| **Marriages** |  | Exogamous with other Muslims (Kashmiri, Gujjar), endogamous (Hindu) | Exogamous with other Muslims (Gujjar) | Exogamous with other Muslims (Pahari), endogamous (Sikh) | Exogamous with other Muslims (Bakarwal) |
