# Supplementary material for: Plant cultural indicators of forest resources from the Himalayan high mountains: implications for improving agricultural resilience, subsistence, and forest restoration
Source: J Ethnobiol Ethnomed. 2024 Apr 24;20:44. doi: 10.1186/s13002-024-00685-w (PMC11040985; doi:10.1186/s13002-024-00685-w)
Supplement: Supplementary file 3 — Additional file 3. List of plant species, local name, part used; life form; preparation, diseases treated, other ethnobotanical uses, traditional cultural use across the four ethnic groups from the Western Himalayas. [file 13002_2024_685_MOESM3_ESM.docx]

**Additional file 2.** List of plant species, local name, part used; life form; preparation, diseases treated, other ethnobotanical uses, traditional cultural use across the four ethnic groups from the Western Himalayas.

| **Name of plant species** |  | **Local name** | **Part used** | **Life from** | **Preparation** | **Diseases treated /**  **Local name of diseases** | **∑U** | **UV** | **IUCN status** | **Other ethnobotanical uses** | **Ethnic groups**  **Frequency of citation/ plant cultural values** | | | |
| --- | --- | --- | --- | --- | --- | --- | --- | --- | --- | --- | --- | --- | --- | --- |
| **Voucher No.** |  |  |  |  |  |  |  |  |  |  | **G** | **B** | **P** | **K** |
| *Abies pindrow* (Royle ex D.Don) Royle  4215-KASH  (Abi.pin)  (Pinaceae) |  | Bunder / Bundul | Bark* | Tree | Bark is boiled in water and the extract obtained is taken orally | Joint pain  Hadi nal dard (G,B,P),  Inflammation  Sujan (P,K,G) | 107 | 0.33 | LC | Wood is used as fire wood and building making | 42 (0.92) | 28 (0.78) | 30 (0.71) | 7 (0.21) |
| *Acer caesium* Wall. ex Brandis  8075-KASH  (Ace.cae)  (Sapindaceae) |  | Chadd / Parth | Seeds and gums(G,B,P) | Tree | Seeds and gums are boiled in water and extract is taken early in the morning | General weakness  Kamzoor (G,B,P) | 82 | 0.25 | LC | Leaves are used as fodder  Stem is used to make agricultural tools. | 36 (0.79) | 32 (0.89) | ------- | 14 (0.73) |
| *Achillea millefolium* L.  4097-KASH  (Ach.mol)  (Asteraceae) |  | Pahelgass | Leaves(B)  **Flowers(K)**  Roots (G) | Herb | Leaves are chewed  **flowers are socked in water for whole day and the extract is taken orally**  10-15 grams of dried root powder is taken orally | Toothache  Dand ko dard (G,B)  **Brain tonic**  Kal doud (K)  Snake bite  Sarfe touf (K) | 91 | 0.28 | LC | Areal part is used as fodder | 26 (0.57) | 25 (0.70) | 40 (0.95) | ------- |
| *Aconitum chasmanthum* Stapf ex Holmes  8045-KASH  (Aco.cha)  (Ranunculaceae) |  | Mohand/ Moori | Roots(B,P) | Herb | Small amount of dried roots are kept under infected teeth | Toothache  Dan nal dard (B,P) | 73 | 0.22 | CR | -------- | ------- | 43 (2.09) | ------- | 30 (0.92) |
| *Aconitum heterophyllum* Wall. ex Royle  4049-KASH  (Aco.het)  (Ranunculaceae) |  | Patris* | Roots*/ leaves (  **Seeds (B)**  G, P) | Herb | Roots are dried and grinded, boiled in water then taken orally or used to cook rice, whichis eaten.  Dried roots are also taken empty stomach in raw form  Paste of leaves is used to treat skin rashes and healing of wounds.  **Seeds are crushed and mixed with honey and applied on throat externally to treat Tonsillitis.** | Back pain  Kamar dard  (B, G).  Anthelminthic  (Malap (G, P),  Neck pain  Gardandard  (P, G,B),  Wound  Zakhm(K),  **Tonsillitis**  Tansor (B) | 184 | 0.56 | CR | ---------- | 65 (1.43) | 39 (1.09) | 50 (1.18) | 30 (0.92) |
| *Aconitum laeve* Royle  7096-KASH  (Aco.lea)  (Ranunculaceae) |  | Moren | Roots (G,P) | Herb | Dried roots are crushed into powder and taken along with water early in the morning | Abdominal pain  Ted dard (G,P) | 68 | 0.20 | LC | ------- | 48 (1.06) | ------- | ------- | 20 (0.61) |
| *Aconitum violaceum* Jacquem. ex Stapf.  8046-KASH  (Aco.cha)  (Ranunculaceae) |  | Pyukha | Roots (K) | Herb | Dried roots kept under tooth to cure toothache  Roots are eaten raw empty stomach | Toothache  Dandas doud (K)  Stomach cramps Peechi (K) | 52 | 0.16 | CR | -------- | ------- | ------ | 52  (1.78) | ------ |
| *Acorus calamus* L.  4119-KASH  (Aco.cal)  (Acoraceae) |  | Vai gunder/ Bach | Roots (G,B) | Herb | Dried roots are taken orally early in the morning.  **Roots are boiled in the water for 20-25 minutes and the extract is taken orally twice a day** | Diarrhoea  Jalab lag gaya (G,B),  Stomach pain  Teda dard (G,B)  **Cough and cold**  Khang (P,G) | 98 | 0.30 | VU | ------- | 58 (1.28) | 40 (1.11) | ------- | ------- |
| *Actaea spicata* var. *acuminata* H.Hara  6242-KASH  (Act.spi)  (Ranunculaceae) |  | Hapat fall | Roots (G,K) | Herb | Dried roots are crushed which are cooked with rice which is taken along with dhesi ghee | Joint pain  Hadi nal dard (G) Aidgan doud (K) | 65 | 0.20 | LC | ------- | 35  (0.99) | ------- | 25  (1.18) | ------- |
| *Adiantum venustum* D.Don  4104-KASH  (Adi.ven)  (Pteridaceae) |  | Kakbi/gauetheer / jail kunji | Frond* | Fern | Extract of whole fern is taken orally.  **Paste of leaves along with mustard oil is applied externally** | Jaundice  Kambda (G,P,B)  **Muscular pain**  Pasli mai dard (P)  Hair fall  Maas narun (K), | 95 | 0.29 | LC | Mature frond is used as toothpick | 25 (0.55) | 15 (0.42) | 45 (1.06) | 10 (0.31) |
| *Adinatum capillus-veneris* L.  4115-KASH  (Adi.cap)  (Pteridaceae) |  | Geautheer / Kunji | Whole plant (K,P) | Herb | Leaves are crushed into powder and mixed with mustard oil and applied externally.  **Whole plant is crushed along with water and kept outside overnight and taken early in the morning** | Swellings  Suj gaye (P),  Hair fall  Maas narun (K)  Bleeding of nose  Naseeir (K) | 95 | 0.29 | LC | Used as toothpick | ------- | ------- | 50 (1.18) | 45 (1.38) |
| *Adonis aestivalis* L.  7100-KASH  (Ado.aes)  (Ranunculaceae) |  | Tank button | Leaves (P,K) | Herb | Paste of the leaves is applied on the infected portions | Skin rashes  Dana nikey (P)  Wound healing  Zakhm (P,K) | 78 | 0.24 | LC | ------- | ------- | ------- | 28 (0.66) | 50 (1.53) |
| *Aesculus indica* (Wall. ex Cambess.) Hook.  4111-KASH  (Aes.ind)  (Sapindaceae) |  | Haandoon/ Mankhhod | Leaves(G) and **seeds** (P) | Tree | Leaves are boiled in the water for 10-20 minutes and the paste is applied externally.  **Oil extracted from dried seed is applied along with mustard oil externally at evening** | Headeach  Sair nal dard (G,P)  Hair fall  Baal pach gayeah (G) | 101 | 0.31 | LC | Leaves are used as fodder | 51 (1.63) | ------- | ------- | 50 (1.53) |
| *Ailanthus altissima* (Mill.) Swingle.  7098-KASH  (Ail.alt)  (Simaroubaceae) |  | Bradey/Bore kul | Bark roots (B) and **leaves** (P) | Tree | Bark is boiled in water to make tea.  **Leaves are crushed to make paste** | Diarrhoea  Julab lag gaya (P)  **Wound healing**  Zakhm (B,P) | 80 | 0.25 | LC | Used as fire wood | ------- | 52 (1.45) | ------- | 28 (1.16) |
| *Ajuga integrifolia* Buch.-Ham. ex D.Don  4243-KASH  (Aju.int)  (Lamiaceae) |  | Jainadum | Leaves (K) | Herb | Leaf extract is taken orally early in the morning twice a day for 2-3 days. | Anthelminthic  Aam (K),  Diarrhoea  Diaherria (K) | 45 | 0.14 | LC | Leaves are used as flavouring agent in milk | ------- | ------- | 45 (1.54) | ------- |
| *Ajuga parviflora* Benth  4095-KASH  (Aju.par)  (Lamiaceae) |  | Jain-a-adam | Whole plant* | Herb | Extract of whole plant is taken empty stomach  Paste of leaves is applied externally | Diarrhoea  Julab (P,G)  Wounds  Zakhm (P,G,K,B) | 165 | 0.50 | LC | ------- | 55 (1.21) | 50 (1.39) | 40 (0.95) | 25 (0.46) |
| *Allium sativum* L.  8118-KASH  (All.sat)  (Amaryllidaceae) |  | Rohan / Thoom | Tuber* | Herb | Tubers are semi burned in the fire and taken orally | High blood pressure  Pressure zada (B,G,P),  Heart diseases  Dilas doud (K)  Asthma  Asthma (K, P) | 148 | 0.45 | LC | Used as food | 25 (0.55) | 15 (0.69) | 18 (0.42) | 90 (2.44) |
| *Allium victorials* L*.*  3812-KASH  (Amarylliaceae) |  | Jungle rohan | Tuber (G,K) | Herb | Dried tuber is crushed into powder and mixed with water and pasted on paper and applied on effected portion | Bone fracture  Zand pach gaye (G)  Fatun (K) | 74 | 0.23 | VU | Leaves are used as vegetable | 44 (0.97) | ------- | 30  (0.71) | ------- |
| *Amaranthus dubius* Mart. ex Thell  7101-KASH  (Ama.dub)  (Amaranthaceae) |  | Ganhar | **Roots** (K) & leaves (G,B) | Herb | Leaves are boiled in water and the extract is used to clean the tooth  **root powder is taken along with milk** | Toothache  Dand doud (K)  Paralysis  Waaj (K), Dangi houna (G,B)  **Weakening of gums**  Thood nal dard (G) | 108 | 0.33 | LC | Leaves are used as vegetable | 22 (0.48) | 58 (1.61) | 33 (0.78) | ------- |
| *Anagallis arvensis* L.  4239-KASH  (Ana.arv)  (Primulaceae) |  | Danddawa | Roots (K) | Herb | Dried roots are rubbed on effected portion | Toothache  Dand doud (K) | 43 | 0.13 | LC | ------- | ------- | ------- | 43 (1.70) | ------- |
| *Androsace rotundifolia* Hardw.  4240-KASH  (And.rot)  (Primulaceae) |  | Uzmpoash | Bulb (G,P) | Herb | Extract from the bulb is mixed with water and taken orally early in the morning | Stomach pain  Maid dard (G,P) | 51 | 0.15 | LC | ------- | 30 (0.99) | ------- | ------- | 21 (1.1) |
| *Anemone falconeri* Thomson  6255-KASH  (Ane.fal)  (Ranunculaceae) |  | Lakutratanjog | Whole plant(B) | Herb | Whole plant is crushed into powder which is taken along with milk or water and taken orally.  **Root powder is applied diretly** | Fever  Taap (B),  Joint pain  Hadi dard (B)  **Wound healing**  Zukam (B) | 45 | 0.14 | LC | ------- | ------- | 45 (1.80) | ------- | ------- |
| *Angelica glauca* Edgew.  4111-KASH  (Ang.gla)  (Apiaceae) |  | Chour/ choro | Roots* | Herb | Root powder is mixed with water and taken orlly early in the morning | Stomach cramps  Marood (G,B,P), Peechi (K) | 85 | 0.26 | EN | Used as spice | 25 (0.55) | 30 (0.83) | 20 (0.47) | 10 (0.31) |
| *Anthemis cotula* L.  4244-KASH  (Ant.cot)  (Asteraceae) |  | FackGass/ mUshki | Whole plant (G,B) | Herb | Extract from the plant is applied externally on effected portion | Insect sting  Lad gaya (G,B),  Muscular pain  Maslin al dard (G,B) | 35 | 0.11 | LC | ------- | ------- | 35 (1.84) | ------- | ------- |
| *Aralia cachemirica* Decne  4245-KASH  (Ara.cac)  (Araliaceae) |  | Khoree | Roots (G,B,P) | Herb | Roots are boiled in water and which is used to cook rice which is taken orally twice a day | Rheumatic pain  Rhe (G,B,P)  Inflammation  Sujan (G)  Gatritis  Maid doud (K) | 114 | 0.35 | VU | Leaves are used as fodder | 45 (1.43) | 45 (1.25) | ------- | 24 (0.73) |
| *Arisaema jacquemontii* Blume  7095-KASH  (Ari.jac)  (Araceae) |  | Hapatgogaj | Tuber (B,P) | Herb | Dried tuber is crushed into powder and applied directly | Burns  Sad gaye (P,B) | 66 | 0.20 | LC | ------- | ------- | 41 (1.69) | ------- | 25 (0.46) |
| *Arisaema propinquum* Schott  7093-KASH  (Ara.pro)  (Araceae) |  | Hapatabij | Tuber (B,P) | Herb | Tuber is crushed which is then applied externally | Blisters  Chala (B,P) | 72 | 0.22 | LC | ------- | ------- | 51 (1.95) | ------- | 21 (0.64) |
| *Arnebia benthamii* Wall. ex G. Don  4096-KASH  (Arn.ben)  (Boraginaceae) |  | Kahzaban/ goazaban | **Leaves** (P,K)  Roots (G,K,P) | Herb | Leaves are boiled in water and and the extaxt is taken orally  **Leaves are added to tea and boiled in water and taken orally**  Roots are crushed into powder and mixed with mustard oil and applied externally. | Hair fall  Bal Girey (G,B,P),  Thirsty  Khuskhi (K)  Cough and cold  Cheas (K),  Neumonia  Neumoonoi (G,P) | 160 | 0.48 | CR | Roots are used against black magic | 50 (0.88) | 75 (1.25) | 20 (0.47) | 15 (0.46) |
| *Artemisia absinthium* L.  4020-KASH  (Art.abs)  (Asteraceae) |  | Teethwan/ TEHTYANO | Whole plant* | Herb | Whole plant extract is used to take early in the morning | Fever  Bukhar (B) Taap (G,P)  Anthelmenthic  Aam (K)  Stomach cramps  Marood (b) | 189 | 0.57 | LC | ------- | 81(1.78) | 40(1.11) | 38(0.47) | 35(1.07) |
| *Artemisia roxburghiana* Besser & Bull  7114-KASH  (Art.rox)  (Asteraceae) |  | Teethwan | Whole plant (B,P) | Herb | Leaves are kept in the water over night and taken orally early in the morning.  Whole plant is boiled in the water and the extract is taken orally twice a day | Stomach pain  Ted dard (B,P)  Anthelminthic  Malap (P)  Fever  Taap (B) | 111 | 0.34 | LC | ------- | ------- | 41(1.14) | ------- | 70 (2.44) |
| *Astragalus grahamianus* Benth.  7102-KASH  (Ast.gra)  (Fabaceae) |  | Krouss | Roots(G,P) | Herb | Small amount of roots is kept under infect teeth | Toothache  Dand nal dard (G,P) | 72 | 0.22 | LC | ------- | 50 (1.10) | ------- | ------- | 22 (0.67) |
| *Atropa acuminata* Royle ex Lindl.  4252-KASH  (Solanaceae) |  | Brand/Methikafile/ chalo lobed | Whole plant (G,B,P) | Herb | **Whole plant is boiled in water of half an hour and the extract is taken orally.**  Leaves are boiled in water for 5-10 minutes and applied externally on effected portion | **Asthma**  Asthma (G.P)  Rheumatic pain  Reh (B) | 84 | 0.26 | EN | ------- | 44 (0.97) | 25 (0.69) | ------- | 15 (0.46) |
| *Berberis aristata* DC.  6247-KASH  (Ber.ari)  (Berberidaceae) |  | Dandledder/ shamloo | Bark(B,P) | Shrub | Bark of the shrub is dried and crushed into powder which is taken along with water or milk | Joint pain  Sang nal dard (B,P) | 97 | 0.29 | CR | Berries are edible Used as fire wood | ------- | 50 (2.50) | ------- | 47 (1.13) |
| *Berberis lyceum* Royle  4102-KASH  (Ber.lyc)  (Berberidaceae) |  | Cxakhmachang/ shamloo | Berries (G,P)  **Bark of roots** (B) | Shrub | Berries are eaten raw.  **Bark of roots is powdered and is applied on affected areas** | Chest pain  Sang nal dard (G,P,B)  **Arthritis**  Reh (P) | 100 | 0.31 | EN | Berries are edible.  Used as fire wood | 55 (1.21) | 15 (0.42) | ------- | 40 (1.22) |
| *Bergenia ciliata* (Haw.) Sternb*.*  4213-KASH  (Ber.cil)  (Saxifragaceae) |  | Pulfort/ butpewa | Roots* | Herb | The root is dried & crushed to make powder is cooked in rice to treat  The powder is mixed with ghee to make it paste which is applied externally | Liver diseases  Kalaji mai dard (G,P)  Rheumatic pain  Reh (K)  Wound healing  Zakhm (B), | 153 | 0.46 | UV | Roots are used to make herbal tea | 53 (1.17) | 60 (1.67) | 40 (0.95) | 10 (0.31) |
| *Betula utilis* D.Don  4015-KASH  (Bet.uti)  (Betulaceae) |  | Burz/ porz | Wood (B,P) | Tree | Glass is made from the trunk of the tree in which water is collected and drinks | Asthma  Asthma (B,P) | 95 | 0.29 | CR | Wood is used as fire wood and bark is used to make tea | ------- | 75 (2.09) | ------- | 20 (0.76) |
| *Bistorta amplexicaulis* (D.Don) Greene 4108-KASH  (Bis.amp)  (Polygonaceae) |  | Manchrai chai/ Masloon | Roots(G,B) | Herb | Roots are used to make tea which is taken orllay twice a day | Whitening of tongue  Zuban kushik (G)  Inflammation  Maidas doud (B)  Fever  Taaf (G) | 111 | 0.34 | LC | Roots are used to make herbal tea | 56 (0.31) | ------- | 55 (1.42) | ------- |
| ***Bistorta amplexicaulis* var*. alba* Munshi & Javeid**  4109-KASH  (Bis.amp.alb)  (Polygonaceae) |  | Manchri chai | Roots(G,B) | Herb | Roots are used to make tea, which is taken early in the morning | Whitening of tongue  Zuban kushki (B)  Stomach cramps  Marood (G) | 97 | 0.29 | LC | Roots are used to make herbal tea | ------- | 45 (1.69) | 52 (1.80) | ------- |
| *Caltha alba* Jacquem ex Cambess  6261-KASH  (Cal.alb)  (Ranunculaceae) |  | Baringu | Roots (G,B) | Herb | Roots are crushed into powder which is taken along with water in the morning | Abdominal pain  Ted dard (G,B) | 68 | 0.21 | LC | ------- | 22 (0.48) | 46 (1.28) | ------- | ------- |
| *Cannabis sativa* L.  4080-KASH  (CAan.sat)  (Cannabinaceae) |  | Bang/ pang | Leaves and  Stem (B,K) | Herb | Powder of leaves and stem is mixed with ghee and taken orally to treat | Skin rashes  Chamdi rati hogaye (B),  Cholera  Cholra (K) | 75 | 0.23 | LC | ------- | ------- | 30 (0.83) | 45 (1.07) | ------- |
| *Capsella bursa pastoris* L.  4250-KASH  (Cap.bur)  (Brassicaceae) |  | Kralmond/ kalmung | Aerial part* | Herb | Paste made from whole plant is applied externally on affected portion | Inflammation  Sujan (B,P),  Wound healing  Zakhm (G,K) | 135 | 0.41 | LC | Leaves are used as vegetable | 35 (0.77) | 15 (0.28) | 25 (0.59) | 55 (1.68) |
| *Cardamine impatiens* L.  6262-KASH  (Car.imp)  (Brassicaceae) |  | Phal-lish | Whole plant (B,P) | Herb | Whole plant extract is taken orally early in the morning. | Fever  Taap (B,P) | 74 | 0.23 | LC | ------- | ------- | 50 (1.39) | ------- | 24 (0.73) |
| *Cuscuta reflexa* Roxb.  4082-KASH  (Cas.ref)  (Convolvulaceae) |  | Kukliport/ fuli | Whole plant (K,P) | Climber | Whole plant is boiled in edible oil and then used externally.  **Herb powder is taken along with water twice a day.** | Joint pain  Hadi nal dard (P)  Wound healing  Zakhm (K,P)  **Asthma**  Asthma (K,P) | 82 | 0.25 | LC | ------- |  |  | 61 (1.44) | 21 (0.64) |
| *Cedrus deodara* (Roxb.) G.Don  4228-KASH  (Ced.deo)  (Pinaceae) |  | Deodar / dair | Stem* | Tree | Oil is extracted by burning resinous wood of stem locally called DEODAR which is applied externally | Toothache  Dand dard (G,K,P)  Lice killing  Jhuu (B) | 158 | 0.48 | LC | Wood is used as fire wood as well as building making, dried resin is eaten raw. | 78 (1.33) | 42 (0.69) | 28 (0.54) | 10 (0.41) |
| *Centaurea iberica* Trevir. & Spreng  4084-KASH  (Cen.ibe)  (Asteraceae) |  | Kreach | Leaves(B) | Herb | Leaves are crushed to make the paste which is applied externally.  Fresh leaves are crushed and the extract is used as eye drop | Burns  Jalna (B)  Eye vision  Lhoo thodi hou gaye (B) | 55 | 0.17 | LC | ------- | ------- | 55 (1.80) | ------- | ------- |
| *Chenopodium foliosum* L.  4085-KASH  (Che.fol)  (Amaranthaceae) |  | Ganhar | **Whole plant** (B,P)  Seeds(K) | Herb | Seeds are boiled in water and mixed with ghee and taken orally.  **whole plant extract is mixed with mustard oil and applied externally** | Joint pain  (Hadi pach gaye (B,P)  **Lice killing**  Jhuu (B,P),  **Hair fall**  Maas narun (K) | 75 | 0.23 | LC | Aerial part is used as fodder | ------- | 25 (0.80) | 23 (1.52) | 22 (0.45) |
| *Cichorium intybus* L.  7115-KASH  (Cic.int)  (Asteraceae) |  | Saz hand/ haend | Whole plant* | Herb | Root powder is mixed with sugar and taken orally twice a day.  Leaves are cooked as vegetable  **Dried herb is powdered and mixed with oil and is applied on effected portions** | Typhoid  Moorkha (P,B),  Blood purifier  Khoon saaf (K),  **Arthritis**  Reh (G) | 163 | 0.49 | LC | Leaves are used as vegetable | 45 (0.99) | 20 (0.56) | 25 (0.59) | 73 (2.23) |
| *Cirsium wallichii* L.  6230-KASH  (Cir.wil)  (Asteraceae) |  | Kund / | Roots (G,P) | Herb | Fresh roots are eaten raw | Abdominal pain  Ted dard (G,P) | 78 | 0.24 | LC | Roots are eaten raw | 42 (0.92) | ------- | ------- | 35 (1.07) |
| *Codonopsis rotundifolia* Benth*.*  6250-KASH  (Cod.rot)  (Campanulaceae) |  | Tundajaid | Whole plant (G,P) | Herb | Whole plant is boiled in water for 25-30 minutes and the extract is taken orally thrice a day | Asthma  Asthma (G,P) | 58 | 0.18 | LC | ------- | 65 (1.43) | ------- | ------- | 33 (1.09) |
| *Colchicum luteum* Baker  6254-KASH  (Col.lut)  (Colchiaceae) |  | Virkumpoash/ Kaymat gul | Tuber *  **Seeds** (G) | Herb | Fresh tuber extract is mixed with ghee then eaten to cure  **Seeds are eaten raw** | Joint pain  Lakh nal dard (G,P)  Cough  Khang (B),  Constipation  Marood (P,G)  **Stomach cramps**  Badhazmi (K) | 117 | 0.35 | EN | Leaves are used as vegetable | 41 (1.47) | 30 (0.83) | 25 (0.59) | 16 (0.49) |
| *Convolvulus arvensis* L.  6252-KASH  (Con.arv)  (Convolvulaceae) |  | Haroli/ baledi | Roots(K,P) | Herb | Decoction of roots is taken twice a day | Constipations  Kabzi (K,P) | 84 | 0.25 | LC | Leaves are used as vegetable | ------- | ------- | 34(1.21) | 50(0.94) |
| *Conyza bonariensis* (L.) Cronquist.  7117-KASH  (Con.bon)  (Asteraceae) |  | Shalut | Leaves | Herb | Leaves are socked into water overnight and taken early in the morning | Dysentery  Choor (K) | 42 | 0.13 | LC | Aerial part is used as fodder | 42 (1.51) | ------- | ------- | ------- |
| *Conyza canadensis* L  4116-KASH  (Con.can)  (Asteraceae) |  | Shalut /kandera | Leaves (G,K) | Herb | Leaves are boiled in water for 10-20 minutes and the extract is taken orally twice a day. | Indigestion  Badhazmi (G)  Stomach gas  Maidas gov gais (K) | 109 | 0.33 | LC | Aerial part is used as fodder | 69 (1.51) | ------- | 40 (0.95) | ------- |
| *Coriandrum sativum* L  6246-KASH  (Cor.sat)  (Apiacae) |  | Dainwall/ dandair | Whole plant* | Herb | Whole plant is crushed into powder and mixed with honey and taken orally twice a day | Hair fall  Charb nal kalaji (P,G)  Fever  Taap (B)  **Piles**  Bawaseir (K) | 138 | 0.42 | LC | Seeds are used as spice | 35 (0.77) | 10 (0.28) | 25 (0.59)   \|  \| \| --- \| | 68 (2.08) |
| *Corydalis govianiana* Wall  3810-KASH  (Cor.gov)  (Fumariaceae) |  | Sang herbi | Roots (B,P) | Herb | Dried roots are crushed into powder and taken along with water twice a day | Respiratory diseases  Sha photi (P),  Chest pain  Sena nal dard (B)  Cough  Khang (P) | 68 | 0.21 | LC | ------- | ------- | 48 (1.33) | ------- | 20 (0.30) |
| *Cydonia oblonga* Mill.  6231-KASH  (Cyd.obl)  (Rosaceae) |  | Bumbcxoonth | Fruit(K,P)  **Seeds** (B,G) | Tree | Fruits are eaten raw mostly in winter season.  **Seeds are boiled in water and used to wash the affected portion** | Cough and cold  Zukam (K,P)  **Boils**  Chaley (G,B) | 135 | 0.42 | VU | Fruits are eaten | 20 (0.44) | 15 (0.42) | 25 (0.60) | 65 (1.97) |
| *Cynoglossum glochidiatum* Wall. ex Benth.  4083-KASH  (Cyn.glo)  (Boraginaceae) |  | Cheur | Roots (K,P) | Herb | Roots of the are crushed into paste which is applied directly | Blisters  Fefad (K),  Boils  Chala (P) | 88 | 0.27 | LC | ------- | ------- | ------- | 58 (1.89) | 30 (1.55) |
| ***Cynoglossum nervosum* Benth.**  7109-KASH  (Cyn.ner)  (Boraginaceae) |  | Neil thoot | Roots(B,P) | Herb | Roots of the are crushed into paste which is applied directly | Jalna (B), Fefad (P) | 52 | 0.16 | LC | ------- | ------- | 25 (0.69) | 27 (1.32) | ------- |
| *Datura stramonium* L.  4085-KASH  (Dat.str)  (Solanaceae) |  | Datuar / Kandero | Seeds (G) leaves (P) | Herb | Seeds are boiled with water and applied externally leaves are boiled in water for 5-10 minutes and applied directly | Frost bites  Shoo (G),  Boils  Chala (P) | 61 | 0.18 | LC | Fruits as used as rodient repellent | 30 (0.59) | ------- | ------- | 31 (1.95) |
| *Delphinium cashmerianum* Royle.  6243-KASH  (Del.cas)  (Ranunculaceae) |  | Moori | **Roots**(G,P,B) | Herb | **Roots are dried and put into a clay made utensil and buried in soil along with honey for 4-5 months which later is taken orally** | **Asthma**  Asthma (G,P,B) | 123 | 0.38 | EN | ------- | 65 (1.43) | 45 (1.25) | ------- | 23 (0.70) |
| *Delphinium roylei* Royle & Munz  7106-KASH  (Del.roy)  (Ranunculaceae) |  | moore | Roots (G,B)  **Flowers** (K) | Her | Dried roots are eaten raw in small quantity early in the morning  **Flowers are collected and used to make khambeer which is eaten mostly in winters** | Anthelminthic  Malap (G,B)  **Cough and cold**  Cheass (K) | 62 | 0.19 | LC | Flowers are used to make jam | 19 (0.42) | 15 (0.42) | 28 (0.59) | ------- |
| *Dioscorea deltoidea* Wall. ex Griseb*.*  6237-KASH  (Dio.del)  (Dioscoreaceae) |  | Single-mingle | Roots(G)  **Leaves** (P) | Climber | Dried roots are crushed and then cooked with rice.  **Juice obtained from leaves is used as eye drops** | Joint pain  Hadi tod (G,P),  **Weak vision**  Looh thodi hou gaye (G,P) | 99 | 0.30 | EN | Roots are used as soap | 69 (1.74) | ------- | ------- | 30 (0.92) |
| *Diplazium maximum* D.Don  7105-KASH  (Dip.max)  (Dryopteridaceae) |  | Deead / Kunji | Young frond* | Fern | Young frond is boiled then dried and cooked as vegetable and used espically in winters | Anthelminthic  Keeday mar (K,P,B)  Asthma  Asthma (G) | 142 | 0.43 | LC | Young frond is used as vegetable | 21 (0.46) | 16 (0.45) | 84 (1.99) | 18 (0.55) |
| *Equisetum arvense* L.  4232-KASH  (Equ.arv)  (Equisetaceae) |  | Gandamgud | Whole plant(B,K) | Fern | Stem is crushed into juice which is taken early in the morning.  Fresh frond is also used to clean teeth. | Kidney stones  Muth nal dard (B),  Teeth cleaning  Dand saaf (K) | 105 | 0.32 | LC | Whole frond is used as brush | ------- | 25 (0.70) | 80 (2.44) | ------- |
| *Equisetum diffusum* D.Don  4231-KASH  (Equ.dif)  (Equisetaceae) |  | Gandamgud / | Whole plant (G,K) | Fern | Stem is crushed into juice which is kept overnight and taken early in the morning.  Fresh frond is also used to clean teeth. | Kidney stones  Muth nal pathri (G) Backwachan kain (K)  Teeth cleaning  Dand dhona (G) | 83 | 0.26 | LC | Whole frond is used as brush | 48  (0.70) | ------- | 35  (0.83) | ------- |
| *Eryngium billardieri*  F.Delaroche  4247-KASH  (Ery.bil)  (Apiaceae) |  | Dawha mool/ Kamna ki jadi | Roots (K) | Herb | Dried roots are taken empty stomach early in the morning | Jaundice  Kambai (K) | 48 | 0.15 | LC | Aerial part is used as fodder | ------- | ------- | 48 (1.13) | ------- |
| *Euphorbia wallichii* Hook.f.  4216-KASH  (Eup.wal)  (Euphorbiaceae) |  | Hearib | Seeds (B) &**stem latex** (G,P) | Herb | **Stem latex is applied externally after cutting the upper portion of the corm.**  Fresh latex is applied externally on effected portion.  Seeds are taken along with piper | **Foot corm**  Bumre (P,G)  Chamdi rati pad gaye (G,B,P),  Cholera  Cholra (P) | 115 | 0.35 | LC | ------- | 35 (0.77) | 55 (1.53) | ------- | 25 (0.76) |
| *Ficus carica* L*.*  4088-KASH  (Fic.car)  (Moraceae) |  | Anjeer | **Fruit** (K) and stem latex (G) | Tree | **Fruits are boiled in water and kept overnight and taken orally early in the morning.**  Stem latex is applied is applied externally. | **Piles**  Bawaseir (K),  Cough and cold Khasi (G),  Skin burns  Chem daze (K) | 149 | 0.45 | LC | Fruits are eaten raw | 75 (1.65) | ------- | ------- | 74 (2.61) |
| *Fragaria nubicola* Lindl. ex Lacaita  4087-KASH  (Fra.nub)  (Rosaceae) |  | Ringrish / maiew | Rhizome(G,P)  Powder(K) | Herb | Tea made of rhizome is taken early in the morning.  Rhizome powder is mixed with water and applied externally | Fever  Kalas doud (K), Taap (G,P),  Tansillitis  Tansol (K) | 127 | 0.39 | LC | Roots are used to make herbal tea | 77 (1.70) | ------- | 32 (0.76) | 18 (0.55) |
| *Fritillaria roylei* Hook.  6238-KASH  (Fri.roy)  (Liliaceae) |  | Sheethkhar | Roots* | Herb | Crushed roots are mixed with water to and taken early in the morning for 2-3 days | Abdominal pain  Ted dard (G,B,P), Maid doud (K),  Rheumatic pain  Reh (G,K) | 182 | 0.55 | CR | ------- | 42  (1.37) | 40  (1.11) | 75  (1.16) | 25  (0.95) |
| *Fumaria indica* (Hausskn.) Pugsley  7111-KASH  (Fum.ind)  (Fumariacaea) |  | Shahtur | Whole plant(P) | Herb | Whole plant extract is taken along with milk. | Joint diseases  Hadi nal dard (P) | 42 | 0.13 | LC | Aerial part is used as fodder | ------- | ------- | 42 (0.99) | ------- |
| *Galium aparine* L.  (Gal.apa)  6248-KASH  (Rubiaceae) |  | Thap gass | Leaves(K,P) | Herb | Whole plant is crushed and applied on effected portion | Wound healing  Zakhm (K,P) | 75 | 0.23 | LC | ------- | ------- | 35 (0.97) | 40 (1.42) | ------- |
| *Gentiana khuroo* Royle  7115-KASH  (Gen.khu)  (Gentianaceae) |  | Nilkanth | Roots (G,B) | Herb | Extract of roots is mixed with water/milk and taken orally | Urine infection  Muth rata pad gaya (G,B) | 68 | 0.21 | CR | ------- | 43 (0.95) | 25 (0.70) | ------- | ------- |
| ***Geranium himalayense*** Klotzsch  7116-KASH  (Ger.him)  (Geraniaceae) |  | Ratanjog | Roots(K,P) | Herb | Root powder is taken along with milk | Rheumatic pain  Reh (K,P) | 58 | 0.18 | LC | Roots are used to prepare herbal tea | ------- | ------- | 48 (1.13) | 10 (0.31) |
| *Geranium pretense* L.  4098-KASH  (Ger.pre)  (Geraniaceae) |  | Ratanjog | Roots* leaves (K) | Herb | Leaves are crushed into powder which is mixed with a glass of water and taken daily for 4 days.  **roots are dried and boiled water is separated which is taken orally or used to cook rice** | Diarrhoea  Choor (K)  Joint pain  Aidgen doud (K), Hadi nal dard (G, B, P)  **General weakness**  Kamzori (G,K,P) | 126 | 0.39 | LC | Roots are used to prepare herbal tea | 86 (1.89) | 15 (0.42) | 20 (0.47) | 5 (0.15) |
| *Geranium wallichianum* Oliv.  4112-KASH  (Ger.wal)  (Geraniaceae) |  | Ratanjog | Roots* | Herb | roots are dried and boiled water and is separated which is taken orally or used to cook rice | Joint diseases  Aidgen doud (K), Hadi nal dard (G, B, P)  General weakness  Kamzori (G,K,P) | 170 | 0.51 | LC | Roots are used to prepare herbal tea | 40 (0.88) | 32 (0.89) | 83 (1.96) | 15 (0.58) |
| ***Geum elatum*** Wall. ex G.Don  7112-KASH  (Geu.ela)  (Rosaceae) |  | Shah buti | Whole plant (G,B,P) | Herb | Whole plant extract is taken early morning along with water | Abdominal pain  Ted dard (G,B, P),  Anthelminthic  Malap (G,P), Keeda (B) | 53 | 0.16 | LC | ------- | 10 (0.95) | 25 (1.39) | 18 (0.71) | ------- |
| *Herniaria hirsuta* L*.*  6241-KASH  (Her.hir)  (Caryophyllaceae) |  | Chikal | **Leaves** (G,B,P) | Herb | **Leaves are grinded and mixed with egg** | **Dizziness**  Chakar aaway (G, P)  Headeach  Sar nal dard (G) | 92 | 0.28 | LC | Leaves are used to make herbal tea | 64 (1.41) | 18 (0.50) | 10 (0.24) | ------- |
| *Hypericum perforatum* L.  4089-KASH  (Hyp.per)  (Hypericaceae) |  | Julab kidawa | Roots (K,G)  Leaves(B) | Herb | Leaves are boiled in water for 10-15 minutes and the extract is taken orally.  roots are dried and boiled water is separated which is taken orally or used to cook rice | Diarrhoea  Julab lag gaya (B)  Joint pain  Aidgen doud (K), Hadi nal dard (G,B) | 103 | 0.32 | LC | Roots are used to prepare herbal tea | 53 (1.17) | 22 (0.61) | 28 (0.66) | ------- |
| *Hyposcyamus niger* L.  4107-KASH  (Hyp.nig)  (Solanaceae) |  | Bazarbang | Whole plant(G,P)  **Seeds** (P) | Herb | Whole plant is crushed into powder and taken orally along with water.  **Seeds are burned to collect the ash which is rubbed on teeth’s** | Joint pain  Hadi dard (P)  **Toothache**  Dand doud (P) | 83 | 0.25 | VU | ------- | 58 (1.28) | ------- | 30 (1.11) | ------- |
| *Impatiens edgeworthii* Hook. f.  7108-KASH  (Imp.edg)  (Balsaminaceae) |  | Buntil | Leaves (B,P) | Herb | Leaves are cooked as vegetable and also applied on effected portion | Skin burns  Jalan (B,P) | 64 | 0.19 | LC | Leaves are used as vegetable | ------- | 30 (0.83) | 34 (1.11) | ------- |
| *Indigofera heterantha* Wall.  8113-KASH  (Ind.het)  (Fabaceae) |  | Zand / gathi | Twigs* | Shrub | Young twigs are used as brush | Cleaning of teeth  Dant saaf* | 95 | 0.29 | LC | Twigs are used as fire wood and also to make wooden buckets | 18 (0.40) | 25 (0.70) | 22 (0.52) | 40 (1.22) |
| *Inula racemosa* Hook. f.  8122-KASH  (Inu.rac)  (Asteraceae) |  | Poshkar mool | Roots(K,B) | Herb | Extract of roots is mixed with sugar and added to milk and taken early in the morning for 2-3 days. | Joint pain  Zang doud (K),  Anthelminthic  Aam (K),  Abdominal pain  Teda dard (B) | 65 | 0.20 | EN | Aerial part is used as fodder | ------- | 65 (1.80) | ------- | ------- |
| *Iris germanica* L.  7118-KASH  (Iri.ger)  (Iridaceae) |  | Mazarmund/ chulindre | Roots (B) | Herb | Powder of dried roots is is taken orally along with water. | Lung diseases  Fefda nal dard (B)  Joint pain  Hadi dard (B) | 44 | 0.13 | LC | Used as rodent repellent in fields | ------- | 44 (2.19) | ------- | ------- |
| *Isodon rugosus* Wall. ex Bentham  6257-KASH  (Iso.rug)  (Lamiaceae) |  | Suliye kath | Leaves (K) and twigs(G) | Shrub | Leaves and twigs are boiled in the water and used to take bath  Extract of leaf is also taken orally | Fever  Kalas doud (K),  Foot fever  Khuran taaf (K),  Diarrhoea  Julab (G), Malap (G) | 118 | 0.36 | LC | Leaves are used to make herbal tea and stem is used as fire wood | 70 (1.51) | ------- | ------- | 48 (1.77) |
| *Juglans regia* L.  7113-KASH  (Jug.reg)  (Juglandaceae) |  | Doon/ khood | Bark* &**seeds** (G,K) | Tree | Bark of the tree is used in cleaning of teeth;  **oil extracted from seeds is applied externally** | Teeth cleaning  Dand saaf (K)  Toothache  Dant douna (G, P),  **Arthritis**  Reh (G,K) | 134 | 0.41 | LC | Fruits are eaten, wood is used as fire wood as well as making furniture | 30 (0.66) | 15 (0.42) | 25 (0.59) | 74 (2.26) |
| ***Juncus inflexus* L.**  7107-KASH  (Jun.inf)  (Juncaceae) |  | Tujj gass | Roots(P) | Herb | Roots are grinded into powder is taken orally with sugar. | Blood in urine  Raat muth na (P) | 40 | 0.12 | LC | ------- | ------- | ------- | ------- | 40 (1.68) |
| *Jurinea dolomiaea* Boiss.  4090-KASH  (Jur.dol)  (Asteraceae) |  | Gogaldoop | Roots* | Herb | Roots are crushed into powder which is taken orally or apllied externally. | Wound healing  Zakhm (G.B.P.K),  Skin burns  Dazun (K), Jalna (G,P) | 172 | 0.52 | VU | Roots are used against black magic, leaves are used to make tea | 48 (1.06) | 82 (2.28) | 30 (0.71) | 12 (0.37) |
| *Lamium alba* L  4092-KASH  (Lam.alb)  (Lamiaceae) |  | Zakhmedawa | Leaves (K) | Herb | Paste of leaves is applied externally on infected portion | Wound healing  Zakhm(K)  Skin burns  Dazun (K) | 48 | 0.15 | LC | Aerial part is used as fodder | ------- | ------- | ------- | 48 (2.08) |
| *Lavatera cashmeriana* Camb.  4099-KASH  (Lav.cas)  (Malvaceae) |  | Jungle sochal | Flowers (K, G) | Herb | flowers are dried and packed in a glass bottle and added with sugar to make jam commonly called khambeer which mostly eaten during winters | Cough and cold  Khang (G), Cheas te sardi (K) | 134 | 0.41 | VU | Flowers are used to make jam | 65 (1.43) | ------- | 65 (1.53) | ------- |
| *Ligularia jacquemontiana* Decne.  4214-KASH  (Lig.jac)  (Asteraceae) |  | Hapat kouth/ | Roots(G,P) | Herb | Dried roots are grinded and taken orally to cure early in the morning | Constipations  Kabzi (G, P),  Anthelminthic  Malap (G,P) | 105 | 0.32 | LC | Leaves are used as fodder | 85 (1.87) | ------- | 20 (0.47) | ------- |
| *Lilium polyphyllum* D.Don  6236-KASH  (Lil.pol)  (Liliaceae) |  | Plean | Tuber (G,P) | Herb | Dried tuber is grinded into powder then cooked with rice and taken along with ghee | **Abdominal bloating**  Naad uthgaye (G, P)  Joint pain  Hadi dard (G,P) | 90 | 0.28 | CR | ------- | 55 (1.21) | ------- | 35 (0.83) | ------- |
| *Linum usitatissimum* L.  8115-KASH  (Lin.usi)  (Linaceae) |  | Alish | Seeds(B) | Herb | Seeds are boiled in water and paste is applied topically | boils  Chala (B),  Joint pain  Tang nal dard (B) | 43 | 0.13 | LC | Leaves are used as fodder | ------- | 43 (1.75) | ------- | ------- |
| *Malva neglecta* Wallr.  4114-KASH  (Mal.neg)  (Malvaceae) |  | Sochal/ jeeli | Whole plant (G,B,P) | Herb | Extract of whole plant is taken orally twice a day | Stomach cramps  Marood (G, P)  Diarrhoea  Julab (B,P) | 135 | 0.41 | LC | Leaves are used as vegetable as well as fodder | 75 (1.65) | 20 (0.56) | 40 (0.95) | ------- |
| *Mentha longifolia* L.  4234-KASH  (Men.lon)  (Lamiaceae) |  | Gud pudine/ chalo purno | Leaves (K,P) | Herb | Leaves are used to make herbal tea  **Whole plant is cooked with egg and taken orally** | Abdominal pain  Ted dard (P)  **Tonsillitis**  Tonsal (K) | 108 | 0.33 | LC | ------- | ------- | ------- | 60 (1.42) | 48 (1.47) |
| *Morina longifolia* Wall.  8126-KASH  (Mor.lon)  (Morinaceae) |  | Kim | Roots (G) | Herb | Small amount of dried roots is taken orally | Anthalmanthic  Malap (G) | 49 | 0.15 | LC | ------- | 49 (1.08) | ------- | ------- | ------- |
| *Nasturtium officinale* W.T.Aiton 4226-KASH  (Nas.off)  (Brassicaceae) |  | Naagsocxal/ Kul haakh | Dried flower* | Herb | Paste of dried flowers is given along with milk | Mumps  Thrass (G,B,K,P) | 145 | 0.44 | LC | Leaves are used to make vegetable | 42 (0.92) | 18 (0.50) | 30 (0.71) | 55 (1.68) |
| *Nepeta cataria* L.  4093-KASH  (Nep.cat)  (Lamiaceae) |  | Gand soi | Leaves (B,K) | Herb | Tea made from leaves is taken early in the morning.  Leaf extract is applied externally | Skin irritations  Chamdi nal khujli (B),  Urine disorders  Peshab khrab(K) | 75 | 0.23 | LC | ------- | ------- | 39 (1.09) | 36 (0.85) | ------- |
| *Nepeta raphanorhiza* Beath.  8127-KASH  (Nep.rap)  (Lamiaceae) |  | Vangogig | Whole plant(P) | Herb | Whole plant extract is taken at bed time | Constipations  Badhazmi (P) | 41 | 0.12 | LC | ------- | ------- | ------- | 41 (1.77) | ------- |
| *Origanum vulgare* L.  4100-KASH  (Ori.vul)  (Lamiaceae) |  | Van Babar/ babre | Whole plant* | Herb | Extract from the plant is taken orally | Skin diseases  Cheam bemair (K).  Intestinal pain  Antadi nal dard (G,P),  Urine disorders  Muuth dard (B) | 139 | 0.42 | LC | Seeds are used as spice | 19 (0.42) | 11 (0.31) | 30 (0.71) | 79 (2.41) |
| ***Oxalis acetosella* L.**  6253-KASH  (Oxa.ace)  (Oxalidaceae) |  | Choxcheng | **Whole plant** (B,P) | Herb | **Whole plant is dried and boiled for 1.-15 minutes and the extract is given as tonic to pregnant women.** | **General weakness**  Kamzoori (B,P) | 98 | 0.30 | LC | Leaves are used to make herbal tea | ------- | 48 (1.89) | 50 (1.42) | ------- |
| *Oxalis corniculata* L.  4113-KASH  (Oxa.cor)  (Oxalidaceae) |  | Choxcheng | Whole plant(G,P) | Herb | Whole plant extract is taken empty stomach for 2-5 days | Diarrhoea  Julab (G,P),  Abdominal pain  Ted dard (G,P) | 79 | 0.24 | LC | ------- | 35  (0.77) | 39  (1.09) | ------- | ------- |
| *Papaver somniferum* L.  8123-KASH  (Pap.som)  (Papaveraceae) |  | Khashkhaash | Stem Latex (K) | Herb | capsule wall powder mixed with water and sugar is taken orally | Stomach pain  Maiddas doud (K),  Diarrhoea  Diahooria (K) | 53 | 0.16 | LC | Seeds are used as flavouring agent in making local bread. | ------- | ------- | ------- | 53 (1  .95) |
| *Parrotiopsis jacquemontiana* (Decne.) Rehder.  6258-KASH  (Par.jac)  (Hamaelidaceae) |  | Poah / pishad | Stem(G,P) &**leaves** (B,K) | Shrub | Oil extracted from stem is applied on affected areas.  **Leaves are crashed into paste and applied externally** | Joint problrms  Aidgen doud (P,K)  **Wound healing**  Zakhm (B.K) | 117 | 0.35 | LC | Twigs are used to make buckets | 34 (0.75) | 31 (0.86) | 29 (0.67) | 23 (0.70) |
| *Phytolacca acinosa* Roxb.  4253-KASH  (Phy.aci)  (Phytolaccaceae) |  | Hapat Braand | Fruits (G) and leaves (P) | Herb | Leaves are crushed along with water to make eye drop. | Eye diseases  Lhoo dhodi hou gaye (G,P) | 135 | 0.42 | LC | Tender leaves are used as vegetable | 75 (0.65) | ------- | 55 (1.30) | ------- |
| *Picrorhiza kurroa* Royle ex Benth*.*  7089-KASH  (Pic.kur)  (Plantaginaceae) |  | Kour | Roots(K) | Herb | Extract of roots is taken twice a day | Stomach pain  Yed doud (K),  Fever  Taaf (K) | 42 | 0.13 | CR | ------- | ------- | ------- | ------- | 42 (1.89) |
| *Pinus wallichiana* A. B. Jacks  4227-KASH  (Pin.wal)  (Pinaceae) |  | Kayur | Resin* | Tree | Resin commonly known as KAANGUL is is applied externally.  Crushed resin along with milk is taken twice a day. | Wound healing  Zakhm (G,B,K,P),  Cracked heels  Khur patun (K)  Influenza  infection (K) | 92 | 0.27 | LC | Wood is used as fire wood and building making | 20 (0.44) | 36 (1.00) | 24 (0.57) | 12 (0.37) |
| *Plantago lanceolata* L.  6249-KASH  (Pla.lan)  (Plantaginaceae) |  | Gul/ niko chemchi pato | Whole plant(K,P) | Herb | Whole plant is crushed along with water and taken orally | Cough and cold  Khang (P),  Asthma  Asthma (K,P) | 128 | 0.39 | LC | Leaves are used as vegetable as well as fodder | ------- | ------- | 68  (1.49) | 60  (2.29) |
| *Plantago major* L*.*  4118-KASH  (Pla.maj)  (Plantaginaceae) |  | Bodd gul/ bado chemchi patro | Whole plant (B,K),  Seeds (B) | Herb | **Decoction of whole plant is taken twice a day.**  Seeds are boiled in water and taken orally.  Fresh leaves are crushed to make paste which is applied externally. | **Abdominal bloating**  Naad (B,K),  Diarrhoea  Julab (B),  Insect sting  Keim douf (K) | 94 | 0.28 | LC | Leaves are used as vegetable as well as fodder | ------- | 40 (1.61) | 54 (1.61) | ------- |
| *Podophyllum hexandrum* (Royle) T.S Ying  4218-KASH  (Pod.hex)  (Berberidaceae) |  | Wanwangun/ jungle ko began | Roots* | Herb | Roots are crushed into powder and taken along with milk for 2-3 days thrice a day. | Tumors  Zarhbad (K),  Diarrhoea  Julab (G.B.P),  Constipations  Kabzi (G.B.P) | 145 | 0.44 | VU | Fruits are eaten raw as well as used against black magic | 23 (0.51) | 25 (0.70) | 82 (1.94) | 15 (0.46) |
| ***Polygonatum biflorum* (Walt.) Ell.**  8120-KASH  (Pol.bif)  (Asparagaceae) |  | Jungli salamesri | Roots (G,P) | Herb | Roots are eaten raw. | Leukorrhea  Mahvari (P,G) | 121 | 0.37 | LC | Tubers are eaten as salad | 71 (1.56) | ------- | 50 (1.18) | ------- |
| *Polygonatum cirrhifolium* (Wall.) Royle  4229-KASH  (Pol.cir)  (Asparagaceae) |  | Salapmesri | Roots (B,P) | Herb | Roots are crushed and mixed with water and taken orally | Leukorrhea (B)  Mahvari (B)  Menstrual disorders (P) | 122 | 0.38 | LC | Tubers are eaten as salad | ------- | 71 (1.98) | 51 (1.21) | ------- |
| *Polygonatum verticillatum* (L.) All.  4230-KASH  (Pol.ver)  (Asparagaceae) |  | Salamesri | Roots* | Herb | Roots are eaten raw as well as after crushed into powder and taken along with milk | Leukorrhea*  Mahvari* | 109 | 0.33 | LC | Tubers are eaten as salad | 35 (0.77) | 16 (0.45) | 46 (1.09) | 12 (0.37) |
| *Polygonium hydropiper* L.  8125-KASH  (Pol.hyd)  (Polygonaceae) |  | Macxrawangan gass/ | Seeds* | Herb | Paste of seeds is mixed with water and applied externally | Whitening of tongue  Khoor kawa hai (G.B.P),  Itchy skin  Kashun (K) | 111 | 0.34 | LC | Leaves are used as vegetable as well as fodder | 25 (0.55) | 15 (0.42) | 60 (1.94) | 11 (0.34) |
| *Polygonium plebeium* R.Br.  8116-KASH  (Pol.ple)  (Polygonaceae) |  | Druab | Leaves (G,K) | Herb | Extract of leaves is taken early in the morning | Pneumonia  Numeni (G)  Stomach pain  Maiyads doud (K) | 77 | 0.23 | LC | Leaves are used as vegetable | 35 (1.65) | ------- | 42 (1.47) | ------- |
| *Potentilla alba* L.  7087-KASH  (Pot.alb)  (Rosaceae) |  | Saban gass | Whole plant(K) | Herb | Whole plant extract is taken orally | Diarrhoea  Choor (K)  Throat swelling  Hatis hunuh (K) | 39 | 0.12 | LC | ------- | ------- | ------- | 39 (1.44) | ------- |
| *Primula denticulata* Sm.  6260-KASH  (Pri.den)  (Primulaceae) |  | Mamera | Roots(G,P) | Herb | Roots are grinded into fine powder and mixed with boiled water after cooling ite used to make eye drops | Weak vision  Lhoo kam (G,P) | 55 | 0.17 | LC | ------- | 30(1.45) | ------- | 25(0.69) | ------- |
| *Prunella vulgaris* L.  4254-KASH  (Pru.vul)  (Lamiaceae) |  | Kallyutt / singota | Whole plant* | Herb | Leaves are boiled in water of 10-15 minutes and applied directly.  Stem extract is boiled in water and taken early in the morning.  Roots are crushed into powder and taken along with water | **Sore throat**  Gala kadwa (G,P)  Cough and cold  Khang (B,P)  Constipations  Badhazmi (G.B.K.P) | 164 | 0.50 | LC | Leaves are used as vegetable as well as fodder | 36 (0.79) | 16 (0.42) | 28 (0.66) | 84 (2.57) |
| *Pteridium revolutum* (Blume) Nakai  7090-KASH  (Pte.rev)  (Dennstaedtiaceae) |  | Jungli Kunji | Young frond* | Fern | Young frond is collected and boiled and dried and crushed into powder and cooked as vegetable | Asthma  Asthma (G,P,K)  Blood pressure  BP (G,P) | 144 | 0.44 | LC | Young frond is used as vegetable | 64 (1.41) | 68 (1.89) | ------- | 12 (0.37) |
| *Rheum webbianum* Royle  4212-KASH  (Rhe.web)  (Polygonaceae) |  | Pambchalan | Roots* | Herb | Crushed roots are mixed with ash and applied externally | Skin diseases  Chem Bemari (K)  Wound healing  Zakhm (B,G,P) | 168 | 0.51 | VU | Leaves are used as vegetable | 33 (0.73) | 80 (2.23) | 30 (0.71) | 15 (0.46) |
| *Rhodoila fastigata* (Hook. f. et Thoms.) Fu  4091-KASH  (Rho.fas)  (Crassulaceae) |  | Hasbi di jadi | Roots (B,P) | Herb | Roots are crushed into powder and taken along with water early in the morning. | Diarrhoea  Julab (B,P) | 67 | 0.20 | LC | ------- | ------- | 46 (1.28) | 21 (0.50) | ------- |
| *Rosa webbiana* Wall. ex Royle  6245-KASH  (Ros.web)  (Rosaceae) |  | Poash / | Flowers* | Shrub | Dried flowers are used to make khambeer which is mostly taken during winters.  **Paste of petals is taken early in the morning along with milk.** | Cough and cold  Khang (G,B,P),  **Chest problems**  Senas doud (K) | 126 | 0.39 | LC | Whole shrub is used as fire wood | 46 (1.03) | 15 (0.42) | 38 (0.90) | 27 (0.82) |
| *Rubia cordifolia* L.  7088-KASH  (Rub.cor)  (Rubiaceae) |  | Thap gass | Roots (K) | Herb | Dried roots are taken in raw form early in the morning | Jaundice  Kambai (K)  Stomach pain  Medas doud (K) | 51 | 0.15 | LC | ______ | ------- | ------- | ------- | 51(1.80) |
| *Rubus ulmifolius* Schott  6235-KASH  (Rub.ulm)  (Rosaceae) |  | Gounch | Whole plant(G,P) | Shrub | Extract from bark and roots are taken orally.  Leaves are crushed into paste and applied externally | Cough and cold  Khang (P),  Diarrhoea  Julab (G,P)  Blisters  Chala (G,B)  Wound healing  Zakhm(G,P) | 72 | 0.22 | LC | Berries are edible | 35 (0.77) | ------- | 37 (0.87) | ------- |
| *Rumex nepalensis* Spreng.  7084-KASH  (Rum.nep)  (Polygonaceae) |  | Abij / olo | Roots* | Herb | Dried roots are crushed and boiled with water and taken orally | Arthritis  Hadi dard (G.B.P)  Asthma  Asthma (G.B.K.) | 143 | 0.43 | LC | Leaves are used as vegetable as well as fodder | 68 (1.50) | 19 (0.53) | 31 (0.90) | 25 (0.76) |
| *Salix alba* L.  7086-KASH  (Sal.alb)  (Saliaceae) |  | Veer kull/ veeso | Twigs* | Tree | Twigs are used to clean teethes,  **juice extracted from young twigs is taken early in the morning** | Teeth cleaning  Dant dhouna (G,P),  **Stomach cramps**  Peechi (K)  **Indigestion**  Badhazmi (G.B.P) | 75 | 0.23 | LC | Leaves are used as fodder and stem is used as fire wood | 15  (0.33) | 5  (0.14) | 30  (0.78) | 25  (0.49) |
| ***Salvia hians* Royle ex Benth**  8115-KASH  (Sal.hia)  (Lamiaceae) |  | Nil-poash | Whole plant (B) | Herb | Whole plant is crushed into powder and kept in mustard oil for a day and the fried and taken orally | Cough and cold  Khang (B)  Defective eye vision  Lhoo pach gaye (B) | 38 | 0.12 | LC | ------- | ------- | 38 (1.53) | ------- | ------- |
| *Sambucus wightiana* Wall.  7091-KASH  (Sam.wig)  (Viburnaceae) |  | Brand | **Roots** (G,B,P) | Herb | **Dried roots are crushed into powder and taken along with milk twice a day for 15-20 days.** | **Polio**  Aingda (B,G,P) | 105 | 0.32 | LC | ------- | 42 (0.92) | 52 (1.45) | 7 (0.17) | ------- |
| *Saussurea costa* (Falc.) Lipsch.  4211-KASH  (Sau.cos)  (Asteraceae) |  | Kouth | Roots* | Herb | Dried roots are boiled and the extract is used used to cook rice, which is taken along with dhesi ghee. | Joint pain  Aidgen doud (K),  Bone fracture  Hadi vich dard (G.B.P) | 179 | 0.54 | CR | Tender leaves are used as vegetable | 25 (0.55) | 89 (2.48) | 47 (1.11) | 18 (0.82) |
| *Selinum vaginatum* C.B.Clarke  3811-KASH  (Sel.vag)  (Apiaceae) |  | Buddjeath/ kratal | Roots* | Herb | Dried roots are taken orally in small quantity along with water. | Stomach pain  Mead doud* | 135 | 0.42 | LC | Aerial part is used as fodder | 16 (0.35) | 78 (2.17) | 26 (0.61) | 15 (0.46) |
| *Senecio chrysanthemoides* DC.  4101-KASH  (Sec.chr)  (Asteraceae) |  | Bouag | Whole plant* | Herb | Paste of whole plant is is applied externally.  **While as decoction of whole plant is taken orally thrice a day** | Scabies  Chem wazlee (K),  Wound healing  Zakhm (G,P)  Abdominal pain  Ted dard (G,B)  **Urine disorders**  Muth khrab (B) | 132 | 0.41 | LC | Leaves are used as vegetable as well as fodder | 36 (0.79) | 41 (1.14) | 31 (0.73) | 24 (0.73) |
| *Silene coronaria* Desr.  4229-KASH  (Sil.cor)  (Caryophyllaceae) |  | Chock dawa | Leaves (G,K) | Herb | Leaves are boiled in the water and then applied externally | Burns  Jalan (G) Dazun (K) | 93 | 0.29 | LC | ------- | 53 (1.17) | ------- | ------- | 40 (1.22) |
| *Silene vulgaris* (Moench) Garcke  8114-KASH  (Sil.vul)  (Caryophyllaceae) |  | Watt kram | Leaves( G,P) | Herb | Leaves are also cooked as vegetable | Abdominal pain  Ted dard (G.P) | 128 | 0.39 | LC | Leaves are used as vegetable | 84 (1.85) | ------- | 43 (1.02) | ------- |
| *Skimmia anquetillia* N.P. Taylor & Airy Shaw  4120-KASH  (Ski.anq)  (Rutaceae) |  | Nair / nairo | Leaves* | Shrub | Leaf extract is mixed with water which is taken orally.  Leaves are boiled in water and the extract is taken orally twice a day.  **Fresh leaves are eaten raw.** | Abdominal pain  Ted dard (G.B.P)  Asthma  Asthma (G,P,K)  **Urine infection**  Muuth khrab (G.B) | 125 | 0.38 | LC | Branches are used as fire wood | 35 (0.78) | 41 (1.14) | 33 (0.78) | 16 (0.49) |
| *Solanum nigrum* L.  8121-KASH  (Sol.nig)  (Solanaceae) |  | Kambai kul | Fruits (K) & leaves(G) | Herb | Leaves are cooked as vegetable.  Fruits are fried and taken orally twice a day | Abdominal pain  Ted dard (G), Yead doud (K),  Jaundice  Kambi (K) | 94 | 0.28 | LC | Leaves are used as vegetable and fruits are eaten raw | 42 (1.37) | ------- | ------- | 52 (1.60) |
| *Sonchus oleraceus* L.  7091-KASH  (Son.ole)  (Asteraceae) |  | Dudij | Leaves (K,P) | Herb | Dried Leaves are grained and boiled in milk and taken at bed time. | Indigestion  Badhazmi (K,P)  Fever  Taap (K,P) | 66 | 0.20 | LC | Leaves are used as vegetable as well as fodder | ------- | ------- | 35 (1.54) | 31 (0.95) |
| *Sorghum halepense* (L.) Pers.  4220-KASH  (Sor.hal)  (Poaceae) |  | Durham / shamkh | Roots (K) | Herb | Paste of roots is mixed with dhesi ghee and applied externally | Dandruff  Kuff (K),  Hair fall  Mass neirun (K) | 40 | 0.12 | LC | ------- | ------- | ------- | ------- | 40 (1.34) |
| *Stellaria media* L.  4249-KASH  (Ste.med)  (Caryophyllaceae) |  | Nick haakh/ | Whole plant* | Herb | Whole plant is crushed along with water to make the paste which is applied externally.  Leaves are cooked as vegetable | Itchy skin  Kashun (K), Khujli (G,B,P)  Pulmonary disorders  Sushas doud (K) | 88 | 0.27 | LC | Leaves are used as vegetable | 19(0.42) | 24(0.67) | 29(0.69) | 16(0.49) |
| ***Swertia petiolata* Royle. ex D.Don**  7094-KASH  (Swe.pet)  (Gentianaceae) |  | Moomrum | Roots (K) | Herb | Dried roots are eaten raw. | Abdominal pain  Yed doud (K) | 38 | 0.12 | LC | ------- | ------- | ------- | 38 (1.37) | ------- |
| *Taraxacum officinalis* (L.) Weber ex F.H.Wigg  6259-KASH  (Tar.off)  (Asteraceae) |  | Heand | Leaves* | Herb | Decoction of leaves is taken orally twice a day.  **Leaves are boiled in water, fried, crushed into paste and applied externally on infected portion** | Stomach cramps  Peechi (K)  Back pain  Kamar dard (G,P)  **Bone fracture**  Tang pach gaye (G.B.P) | 175 | 0.53 | LC | Leaves are used as vegetable | 42 (0.70) | 18 (0.50) | 41 (0.97) | 75 (2.60) |
| *Thymus linearis* Benth  4107-KASH  (Thy.lin)  (Lamiaceae) |  | Javind/ Naimun/ jemain | Leaves* | Herb | Leaves are boiled with water and added with eggs  **Extract of leaves is used as eye drop** | Stomach cramps  Marood (G,B, P).  **Weak vision**  Gash kam (K) | 128 | 0.39 | LC | Leaves are used to make herbal tea | 16 (0.35) | 11 (0.31) | 85 (2.01) | 15 (0.55) |
| *Trigonella foenum-graecum* L.  4248-KASH  (Tri.foe)  (Fabaceae) |  | Meath/ meethi | Leaves (G,P) & seeds (K,B) | Herb | Seeds are grinded into powder then mixed with egg yolk then pasted on a paper and applied externally;  Leaves are socked in water and kept overnight and taken early in the morning | Bone fracture  Tang pach gaye (G,B,K),  Abdominal pain  Mead doud (P)  Intestinal pain  Andram doud (K) | 134 | 0.41 | LC | Seeds are used as spice | 24 (0.53) | 10 (0.28) | 17 (0.40) | 83 (2.54) |
| *Tulipa clusiana* Red.  7097-KASH  (Tul.clu)  (Liliaceae) |  | Yerki poash | Tuber (K) | Herb | Dried tubers are crushed into powder and boiled in milk for 10-20 minutes and taken orally. | General weakness in pregnant women  Kamoori Loos ziyan (K) | 48 | 0.15 | LC | ------- | ------- | ------- | 48 (1.34) | ------- |
| *Tussilago farfara* L.  4103-KASH  (Tus.far)  (Asteraceae) |  | Watpan | Roots (G,B,P) | Herb | Roots are taken raw early in the morning | Pulmonary diseases  Fefda di bemari (G,P),  Abdominal pain Ted dard (G,B) | 72 | 0.22 | LC | ------- | 38  (0.84) | 14  (0.40) | ------- | 20  (0.61) |
| *Urtica dioica* L.  4219-KASH  (Uri.dio)  (Urticaceae) |  | Soi/ kaire | **Roots** *  Leaves (K) | Herb | **Paste of roots mixed with oil is applied externally**  Root is boiled in water and taken early in the morning  Paste of leaves is applied externally | **Joint pain**  Aidgen doud (K),  Wound healing  Zakhm (G,B,K,P),  Asthma  Asthma (P,B) | 103 | 0.32 | LC | Leaves are used as vegetable | 23 (0.51) | 30 (0.83) | 28 (0.73) | 18 (0.55) |
| *Valeriana jatamansi* Jones  4237-KASH  (Val.jat)  (Valerinaceae) |  | Mushkbalay | Whole plant* | Herb | Whole plant is crushed along with water and the paste is applied on effected portions | Fever  Taap (G,B,P)  Headache  Kalas doud (K). | 82 | 0.25 | LC | ------- | 20 (0.44) | 29 (0.81) | 21 (0.49) | 12 (0.37) |
| *Verbascum thapsus* L  4242-KASH  (Ver.tha)  (Scrophulriaceae) |  | Sarfe makai/ Vantamookh | Seeds (K) &leaves(G,P) | Herb | Seeds are boiled in the water and the extract is taken orally.  Paste of leaves is applied externally. | Asthma  Asthma (G.K.P)  Skin burns  Dazun (K) | 99 | 0.30 | LC | Aerial part is used as fire wood | 29 (0.64) | ------- | 40 (0.95) | 30 (0.92) |
| *Verbena officinales* L  4117-KASH  (Vib.off)  (Verbenaceae) |  | Hatmool | Whole plant(B) | Herb | **Whole plant extract is taken along with water.**  Fresh leaves are crushed into paste and applied externally. | **Indigestion**  Badhazmi (B),  Wound healing  Zakhm (B) | 66 | 0.20 | LC | Areal part is used as fodder | ------- | 16 (0.45) | 40 (0.95) | ------- |
| *Veronica persica* Poir.  7104-KASH  (Ver.per)  (Plantaginaceae) |  | Poeatkaich | Whole plant (G) | Herb | Dried plant is crushed into powder which is mixed with mustard oil and applied externally | Dermatitis  Kushki (G) | 41 | 0.12 | LC | Leaves are used as vegetable | 41 (1.52) | ------- | ------- | ------- |
| *Viburnum grandiflorum* Wall. ex DC  4241-KASH  (Vib.gra)  (Viburnaceae) |  | Kilmish/ Kulmach/ gouch | Roots (K,G)  **Seeds** (B) | Shrub | Roots are boiled in water and then taken with food to treat.  **Seeds are boiled in water for 20-25 minutes and the extract is taken thrice a day for 5 days.** | Cough and cold  Cheas (K,G)  **Stomach pain**  Medas doud (B), | 109 | 0.33 | LC | Fruits are eaten raw, branches are used to make buckets | 39 (0.86) | 24 (0.67) | 31 (0.73) | 15 (0.46) |
| *Viola biflora* L.  8120-KASH  (Vio.bif)  (Violaceae) |  | Gul nakash | Whole plant (G,P) | Herb | Extract of flowers and used is mixed with oil and applied externally.  **Dried leaves are boiled in water and the extract is taken orally after cooling.** | Joint pain  Hardi dard (G,P),  **Fever**  Taap (G,P) | 75 | 0.23 | LC | ------- | 51  (1.12) | ------- | 24  (0.57) | ------- |
| *Viola odorata* L.  4238-KASH  (Vio.odo)  (Violaceae) |  | Nunpoash | Whole plant (B,P) | Herb | Flowers are collected in early spring and dried up to make Khambeer which is taken orally in winter. | Cough and cold  Khang (B,P), Sardi (P) | 94 | 0.29 | LC | ------- | ------- | 49 (1.36) | 45 (1.06) | ------- |
| *Viscum album* L*.*  8119-KASH  (Vis.alb)  (Santalaceae) |  | Alhatch / muneen | Aerial part (K) | Shrub | Aerial part is dried and crushed into powder and taken along with water twice a day. | Joint pain  Aidgen doud (K) | 53 | 0.16 | LC | Seeds are used as glue also used as fodder | ------- | ------- | 53 (1.25) | ------- |
| *Xanthium spinosum* L.  4241-KASH  (Xan.spi)  (Asteraceae) |  | Chear ghass | Roots(G,P) | Herb | Roots are dried and crushed into powder and taken early in the morning | Stomach pain  Meda dard (G,P) | 76 | 0.23 | LC | Fruits are used as rodent repelant | 46 (1.01) | ------- | ------- | 30 (0.61) |

Abbrevations used: CR- Critically endangered; EN – Endangered; VU- vulnerable; LC- least concern; **bold represents novel uses of species.**
